# Phenotype-Genotype Association Analysis of ACTH-Secreting Pituitary Adenoma and Its Molecular Link to Patient Osteoporosis

**DOI:** 10.3390/ijms17101654

**Published:** 2016-09-29

**Authors:** Renzhi Wang, Yakun Yang, Miaomiao Sheng, Dechao Bu, Fengming Huang, Xiaohai Liu, Cuiqi Zhou, Congxin Dai, Bowen Sun, Jindong Zhu, Yi Qiao, Yong Yao, Huijuan Zhu, Lin Lu, Hui Pan, Ming Feng, Kan Deng, Bing Xing, Wei Lian, Yi Zhao, Chengyu Jiang

**Affiliations:** 1 Department of Neurosurgery, Peking Union Medical College Hospital, Chinese Academy of Medical Sciences, Peking Union Medical College, Beijing 100730, China; yangyakun2004@163.com (Ya.Y.); liuxiaohai09@126.com (X.L.); daicongxinxin@163.com (C.D.); bw_sun@163.com (B.S.); qiaoyipumch@126.com (Y.Q.); yaoyongpumc@163.com (Yo.Y.); fengmingpumch@126.com (M.F.); dengkanpumch@126.com (K.D.); xingbingpumch@126.com (B.X.); lianweipumch@126.com (W.L.); 2 Center for Translational Medicine, Peking Union Medical College Hospital, Chinese Academy of Medical Sciences, Peking Union Medical College, Beijing 100730, China; 3 Joint Pituitary Research Center of Cedars-Sinai Medical Center & Peking Union Medical College Hospital, Beijing 100730, China; cuiqi.zhou@cshs.org; 4 State Key Laboratory of Medical Molecular Biology, Institute of Basic Medical Sciences, Chinese Academy of Medical Sciences; Department of Biochemistry and Molecular Biology, Peking Union Medical College, Tsinghua University; Beijing 100005, China; shengmm@aliyun.com (M.S.); hfmyanyan@gmail.com (F.H.); zhujindong1990@sina.com (J.Z.); 5 Key Lab of Intelligent Information Processing of Chinese Academy of Sciences (CAS), Institute of Computing Technology, CAS, Beijing 100190, China; dechao.bu@gmail.com; 6 Department of Medicine, Cedars-Sinai Medical Center, Los Angeles, California 90048, USA; 7 Department of Endocrinology, Peking Union Medical College Hospital, Chinese Academy of Medical Sciences, Peking Union Medical College, Beijing 100730, China; zhuhuijuanpumch@126.com (H.Z.); lulinpumch@126.com (L.L.); panhuipumch@126.com (H.P.)

**Keywords:** Adrenocorticotrophin (ACTH)-secreting pituitary adenoma, phenotype, genotype, osteoporosis

## Abstract

Adrenocorticotrophin (ACTH)-secreting pituitary adenoma, also known as Cushing disease (CD), is rare and causes metabolic syndrome, cardiovascular disease and osteoporosis due to hypercortisolism. However, the molecular pathogenesis of CD is still unclear because of a lack of human cell lines and animal models. Here, we study 106 clinical characteristics and gene expression changes from 118 patients, the largest cohort of CD in a single-center. RNA deep sequencing is used to examine genotypic changes in nine paired female ACTH-secreting pituitary adenomas and adjacent nontumorous pituitary tissues (ANPT). We develop a novel analysis linking disease clinical characteristics and whole transcriptomic changes, using Pearson Correlation Coefficient to discover a molecular network mechanism. We report that osteoporosis is distinguished from the phenotype and genotype analysis. A cluster of genes involved in osteoporosis is identified using Pearson correlation coefficient analysis. Most of the genes are reported in the bone related literature, confirming the feasibility of phenotype-genotype association analysis, which could be used in the analysis of almost all diseases. Secreted phosphoprotein 1 (*SPP1*), collagen type I α 1 chain (*COL1A1*), 5′-nucleotidase ecto (*NT5E*), HtrA serine peptidase 1 (*HTRA1*) and angiopoietin 1 (*ANGPT1*) and their signalling pathways are shown to be involved in osteoporosis in CD patients. Our discoveries provide a molecular link for osteoporosis in CD patients, and may open new potential avenues for osteoporosis intervention and treatment.

## 1. Introduction

Adrenocorticotrophin (ACTH)-secreting pituitary adenomas, also called Cushing disease (CD), is a rare but aggressive endocrine disorder [1]. The incidence of ACTH-secreting pituitary adenomas was reported as approximately 0.8–2.4 cases per million in the general population worldwide, whereas its population prevalence was estimated at 20–32 cases per million [2,3]. Interestingly, 80%–90% of ACTH-secreting pituitary adenomas occur in females aged 35–44 years. Cushing’s disease accounts for approximately 80% of cases of Cushing’s syndrome (CS) [4]. Clinical characteristics often include metabolic syndrome, heart disease, osteoporosis and others [5]. Currently, transsphenoidal surgery is the primary treatment choice for ACTH-secreting pituitary adenomas in addition to oral drugs and radiation therapy [6,7]. Unfortunately, both in terms of the percentage of patients who do not achieve postoperative endocrine remission, and the percentage of disease recurrence after transsphenoidal surgery, remain high [8]. Studies showed that the risk of recurrence after surgery persisted for up to 10 years, and the rate of long-term failure of pituitary surgery was approximately 32% [9]. Nearly one third of patients required an additional second-line treatment, including second surgery, radiotherapy, bilateral adrenalectomy and medical therapy. However, each approach has its limitations [10]. Therefore, it is critical to understand molecular mechanisms of CD and elucidate intrapituitary hormone networks.

ACTH-secreting pituitary adenomas promote excess adrenal cortisol production. Excess cortisol affects the metabolism through a variety of mechanisms including the redistribution of free fatty acids to central fat, increasing gluconeogenesis, the inhibition of glucose uptake by peripheral tissues and impairing insulin action [11,12]. These disturbances lead to an increased risk of cardio- and cerebro-vascular disease [13]. A total of 50%, 93% and 56% of patients have diabetes mellitus, hypertension and dyslipidaemia, respectively [14]. Structural and functional impairment of the skeletal system is commonly encountered and leads to disability in CD patients. Approximately 30%–50% of patients with CS experience fractures, particularly at the spinal level, consistent with 50% of incidence of osteoporosis [15]. Excess cortisol disrupts the bone collagenous matrix, reduces its synthesis and also increases its degradation [16]. Decreasing the number and function of osteoblast plays a central role in bone loss, secondary to excess cortisol [17]. Although previous reports have described subcellular disruptions in CD [18,19], the full understanding of the disease’s molecular network using whole Omics technology has not been established. The relationship between the clinical characteristics and gene expression changes in ACTH-secreting pituitary adenomas has not been reported.

Here, we report that osteoporosis is distinguished from the analysis of 106 clinical characteristics and gene expression changes from 118 patients. The molecular mechanism of osteoporosis in CD patients is elucidated. *SPP1* (secreted phosphoprotein 1), *COL1A1* (collagen type I α 1 chain), *NT5E* (5′-nucleotidase ecto), *HTRA1* (HtrA serine peptidase 1) and *ANGPT1* (angiopoietin 1) and their signalling pathways involving osteoporosis in CD patients are identified.

## 2. Results

### 2.1. Clinical Characteristic Association Analysis

**Figure 1:**
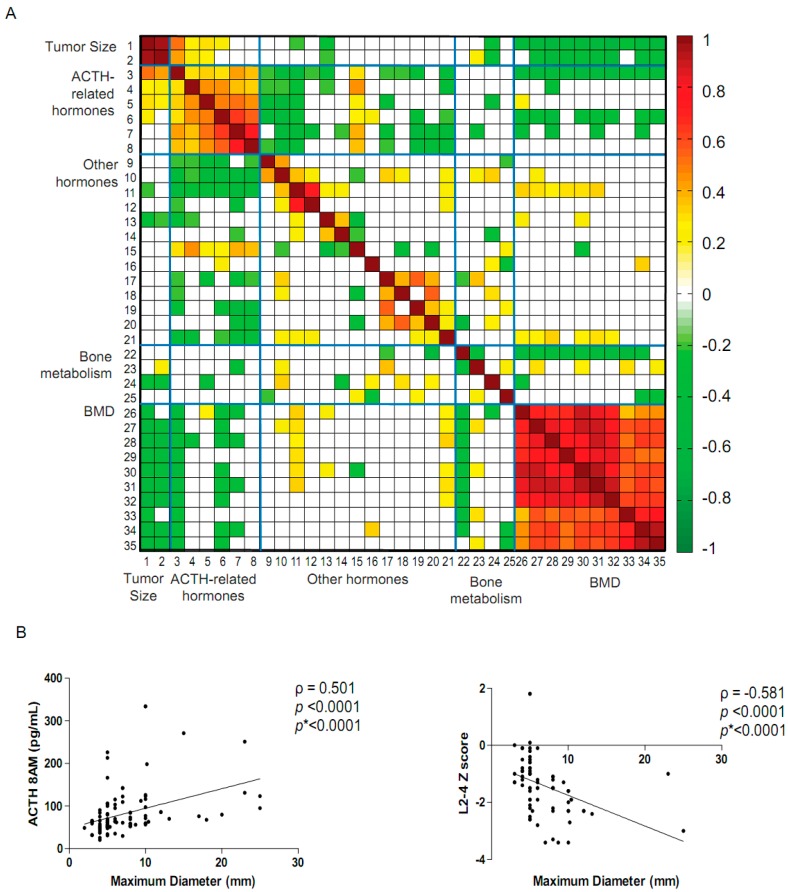
Adrenocorticotrophin (ACTH)-secreting pituitary adenoma tumor size is correlated with pituitary-related hormones and bone mineral density parameters. (**A**) 35 clinical characteristics indices were processed for Spearman’s rank correlation coefficient (SRCC) analysis. These phenotypes were shown in Table S1. The heat map of the SRCC results (*p* value < 0.05, false discovery rate (FDR) < 0.05) were generated using Matlab 7.0 software (WathWorks, Natick, MA, USA). Warm colours represent positive correlations, and cold colours represent negative correlations. White indicates no association; (**B**) The Maximum tumor diameter was positively correlated with the 8 Ante Meridiem (AM) plasma ACTH level and was negatively correlated with age-matched bone mineral density (BMD) indices L2–4. Spearman’s rank correlation analysis (ρ), the *p* value and the Benjamini-Hochberg multiple testing correction (*p* *) were used to control for the false discovery rate (FDR) and were provided in each graph.

We performed a transsphenoidal surgical resection on 118 ACTH-secreting pituitary adenomas over a period of 14 months (Figure S1, Table 1). This series of an infrequent endocrine tumor was derived from a catchment of more than 100 million Chinese inhabitants. We first analysed 106 clinical characteristics using Spearman’s rank correlation coefficient analysis. We selected the related coefficient of each of the two phenotypes that met the requirements of multiple testing corrections and adjusted *p*-values < 0.05. Calculated results were displayed as a heatmap picture using Matlab version 7.0 (Figure 1 and Figure S2, Table S1). We found that pituitary tumor size was correlated linearly with levels of ACTH, cortisol, luteinizing hormone (LH) and estradiol (E2) as well as clinical characteristics of bone metabolism and bone mineral density (BMD). However, there was a poor correlation with the clinical characteristics of the routine blood chemistries, cardiovascular related indices, liver function, electrolyte, renal function, lipid metabolism or glycometabolism (Figure 1 and Figure S2, Table S1).

The maximum ACTH-secreting pituitary adenoma diameter was positively correlated with the blood ACTH levels and both serum and urinary cortisol (Figure 1 and Figure S3, Table S2), and inversely linearly correlated with all ten age-matched BMD parameters (Z scores), thus larger tumor size was associated with lower BMD Z scores (Figure 1 and Figure S3, Table S2).

Moreover, ACTH and cortisol had a moderate inverse correlation with ten BMD Z scores, which was consistent with previous reports [20] (Figure 1, Figures S4 and S5, Tables S3 and S4). Interestingly, LH and E2 levels, which were inversely associated with tumor size, were moderately positively linearly correlated with only the lumbar of age-matched BMD parameters (Z scores) (Figure 1, Figures S6 and S7, Tables S5 and S6), which were consistent with the previously demonstrated protective roles of LH and E2 on bone fracture [21]. Taken together, these results suggest that tumor size and increased ACTH and cortisol level contribute to the high incidence of osteoporosis in ACTH-secreting pituitary adenoma patients [22].

### 2.2. Gene Expression Profile of Adrenocorticotrophin (ACTH)-Secreting Pituitary Adenomas

To gain further insights into the molecular pathogenesis of ACTH-secreting pituitary adenomas, RNA deep sequencing was used to examine genotypic changes in nine paired female ACTH-secreting pituitary adenomas and adjacent nontumorous pituitary tissues (ANPT) (Table S7). A total of 31,933 of 32,186 protein-coding Refseq genes were expressed in ACTH-secreting pituitary adenomas and their matched controls (FPKM (fragments per kilobase of exons per million mappable reads) > 0) [23,24]. Overall, 423 genes (153 genes up-regulated and 270 genes down-regulated, fold change > 2.5) were identified to be significantly differentially expressed and were selected for subsequent analyses.

**Figure 2:**
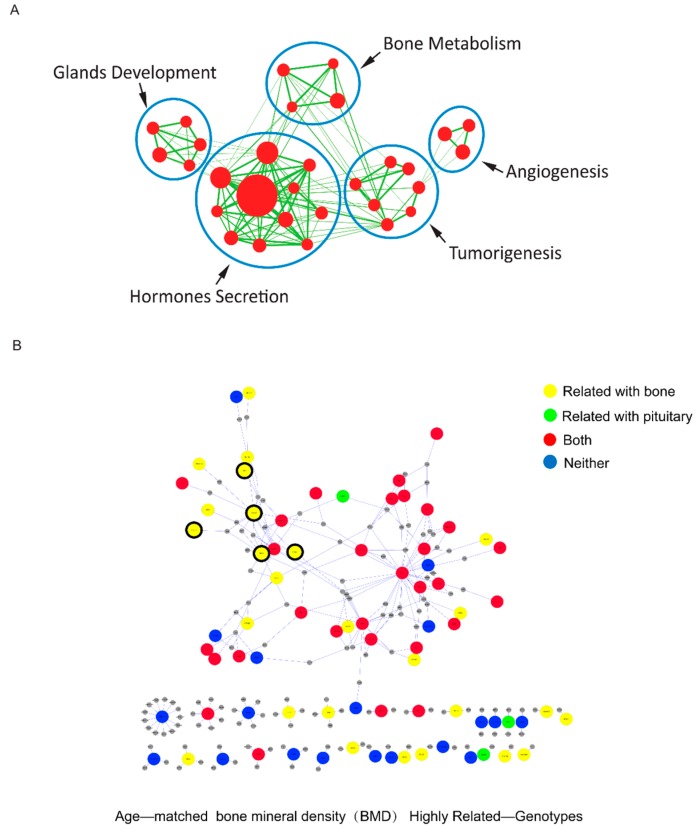
Gene expression profile of ACTH-secreting pituitary adenomas. (**A**) The 423 differentially expressed protein-coding genes in ACTH-secreting pituitary adenoma for the functional enrichment test, GO (Gene Ontology) terms are presented using Cytoscape software (National Institute of General Medical Sciences, USA). Each node is a gene set. The node size is proportional to the number of differentially expressed and interacting genes for this term; the edge thickness represents the number of overlapping genes between sets; (**B**) Literature mining of 138 highly related BMD genes in protein-protein interactions (PPI) connection. Red represents genes reported in the literature for both bone and pituitary adenoma disorders. Yellow and green represent genes reported for bone or pituitary adenomas field, respectively. Blue represents genes not previously reported for bone nor pituitary adenoma fields. The black frame represents the five selected genes.

Thus, 423 differentially expressed genes were studied by functional gene enrichment analysis [25]. The Cytoscape software was used to illustrate the GO (Gene Ontology) terms functional enrichment test. Each group (node) is a gene set, in the biological process of GO terms analysis. We were intrigued to discover that most of the significantly differentially expressed genes were clustered into five groups: tumorigenesis, angiogenesis, gland development, hormone secretion and bone metabolism (Figure 2A). These genotype enrichment results were highly consistent with the observed clinical phenotype changes.

To further identify the genes responsible for these phenotypic changes, we examined the correlations of 10 BMD parameters with the 423 robustly differentially expressed genes using a Pearson coefficient correlation (PCC) analysis. There were 138 significant differential expression genes which were related to BMD parameters, these genes were then subjected to a PubMed for literature mining. The functions of 40 of the 138 bone-related genes had previously been reported for both bone and pituitary adenoma. The function of 39 genes had been reported only for bone disorders, while the functions of eight genes had been reported to be relevant to pituitary adenomas. Overall, 79 of 138 bone-related genes analysed in our study have been previously reported as related to bone disorders, suggesting that our strategic analysis is relevant and pragmatic. The 51 unreported genes may have novel functions involved in osteoporosis induced by ACTH-secreting pituitary adenomas. Further experimental studies are necessary to confirm this hypothesis.

We used the human protein-protein interactions (PPI) database to construct a network with these 138 genes. Only 78 protein interactions derived from these 138 genes existed in the database and were therefore selected. These 78 genes were selected and mapped to the protein-protein interaction network, and the sub-network was then visualised by cytoscape (Figure 2B and Figure S8, Tables S8 and S9). Similar results were found for genes highly related to ACTH 8 AM, 24 h urinary-free cortisol, LH and E2 (Figure S9, Tables S10–S13). Notably, 48 of 78 genes visualised by the PPI cytoscape formed one network, while the remaining 30 genes were not connected (Figure 2B). This result suggests that the network formed with 48 differentially expressed genes in ACTH-secreting pituitary adenomas may reveal the molecular mechanism of CD-related osteoporosis.

### 2.3. Molecular Link to Patient Osteoporosis

**Figure 3:**
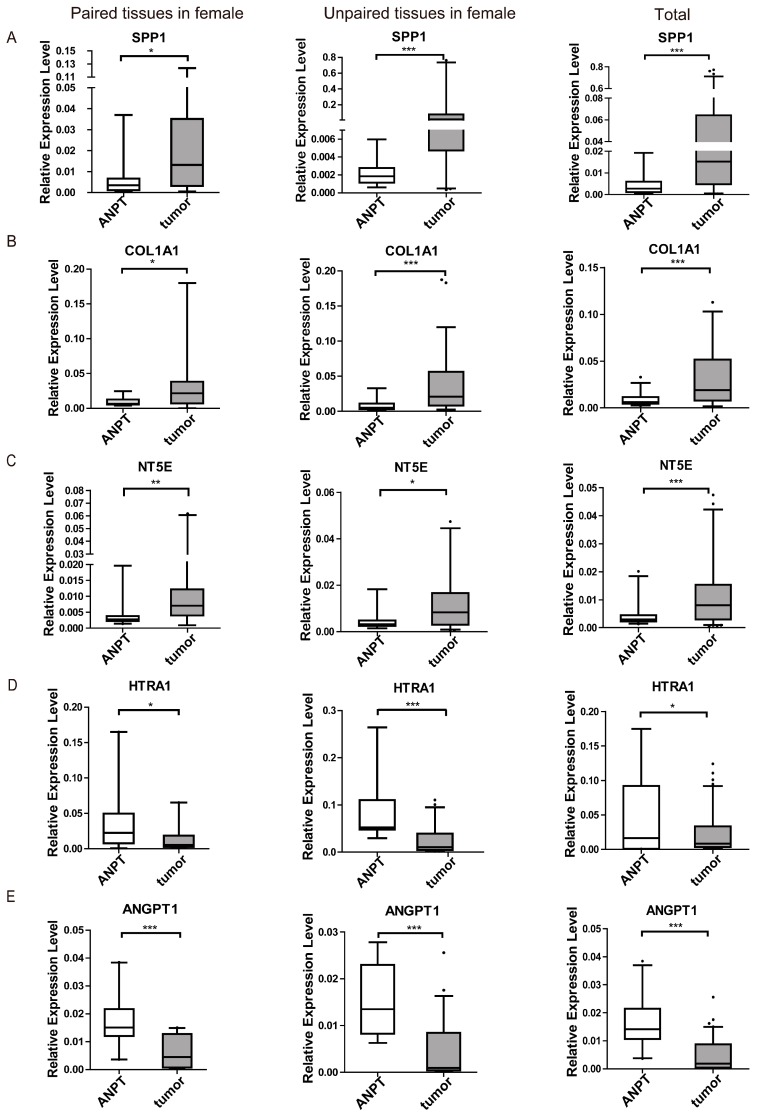
Gene expression profile of ACTH-secreting pituitary adenomas using qRT-PCR. A comparison of expression levels of *SPP1* (**A**), *COL1A1* (**B**), *NT5E* (**C**), *HTRA1* (**D**) and *ANGPT1* (**E**) between ANPT and ACTH-secreting pituitary adenomas using qRT-PCR including 29 female paired pituitary tissues, 63 female unpaired tissues (ANPT, *n* = 13; tumor, *n* = 50) and 109 total tissues (female, *n* = 92; male, *n* = 17). The data are representative of three technical repeats in the median (quartiles) (Mann-Whitney test, * *p* < 0.05, ** *p* < 0.01, *** *p* < 0.001. ANPT versus tumors).

Of 48 differentially expressed genes in the network, 15 are associated with bone functions in the literature mining (Figure 2). We selected five genes, *SPP1*, *COL1A1*, *NT5E*, *HTRA1* and *ANGPT1*, of 15 genes whose coding proteins could be secreted from the pituitary, and we examined their tumor expression in 118 CD patients. In the RNA-seq data of nine CD patients, the *SPP1*, *COL1A1* and *NT5E* gene expression levels were elevated in tumors compared with ANPT, while the *HTRA1* and *ANGPT1* gene expression levels were down-regulated (Table S14). We used quantitative RT-PCR (real-time polymerase chain reaction) to examine the expressions of these five genes in an additional panel of 109 ACTH-secreting pituitary adenomas obtained from three validation groups: Group 1 consisted of 29 female paired samples of ACTH-secreting pituitary adenomas and their respective ANPT controls; Group 2 consisted of 63 female ACTH-secreting pituitary adenoma including 50 ACTH-secreting pituitary adenoma tissues and 13 ANPT controls; Group 3 consisted of 13 male ACTH-secreting pituitary adenoma tissues and four male ANPT controls. The expression of the five genes was significantly different between the ACTH-secreting pituitary adenoma tissues and ANPT controls (Figure 3 and Figure S10). These differences in gene expression were consistent with their respective RNA-seq data.

**Figure 4:**
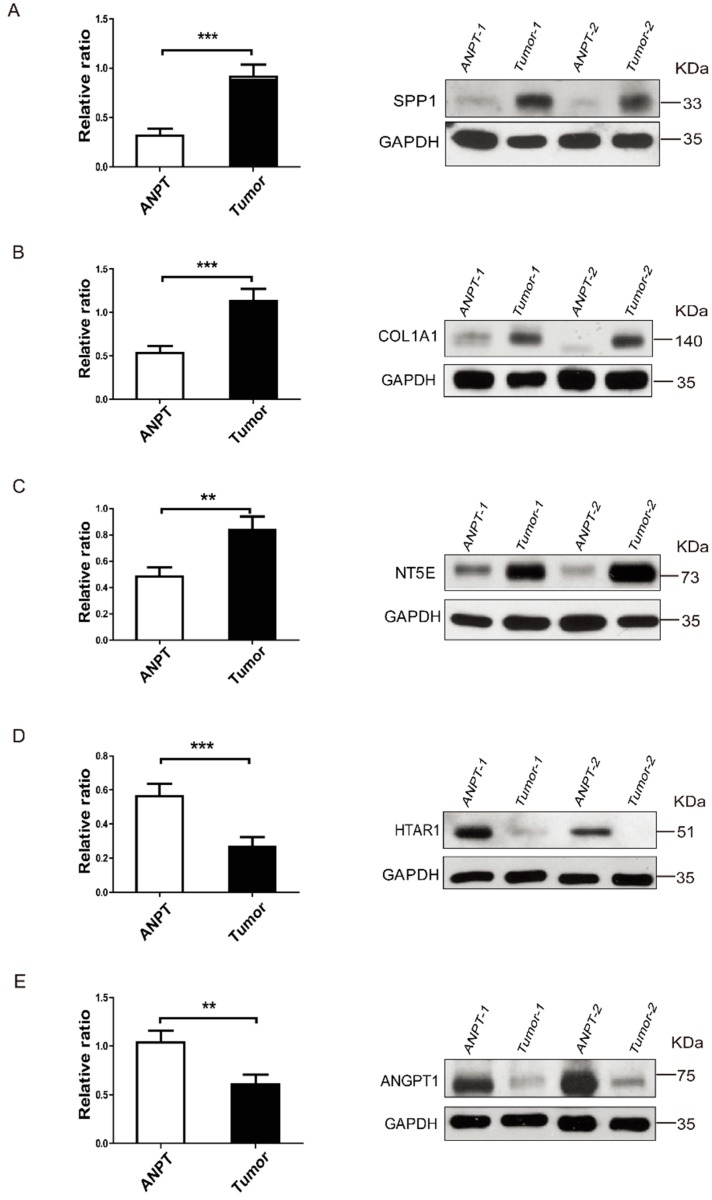
Gene expression profile of ACTH-secreting pituitary adenomas using western blot assay. SPP1 (**A**), COL1A1 (**B**), NT5E (**C**), HTRA1 (**D**) and ANGPT1 (**E**) protein levels in ANPT and ACTH-secreting pituitary adenomas are assessed using western blots in 29 female paired pituitary tissues. Bar graph shows the SPP1/GAPDH relative ratio, COL1A1/GAPDH relative ratio, NT5E/GAPDH relative ratio, HTRA1/GAPDH relative ratio and ANGPT1/GAPDH relative ratio (ANPT *n* = 29, tumors *n* = 29). The data are representative of three technical repeats with the mean ± SEM (two-tailed Student’s *t*-test, ** *p* < 0.01, *** *p* < 0.001. ANPT versus tumors).

We also measured the expression levels of five proteins from Group 1 specimens by Western blot assay. As was consistent with the qRT-PCR results, SPP1, COL1A1 and NT5E proteins were up-regulated, while the expression of HTRA1 and ANGPT1 were down-regulated in the tumor compared with ANPT groups (*n* = 29, Figure 4 and Figure S11). Taken together, these five differentially expressed genes in CD tumors may play important roles in CD-related osteoporosis.

**Figure 5:**
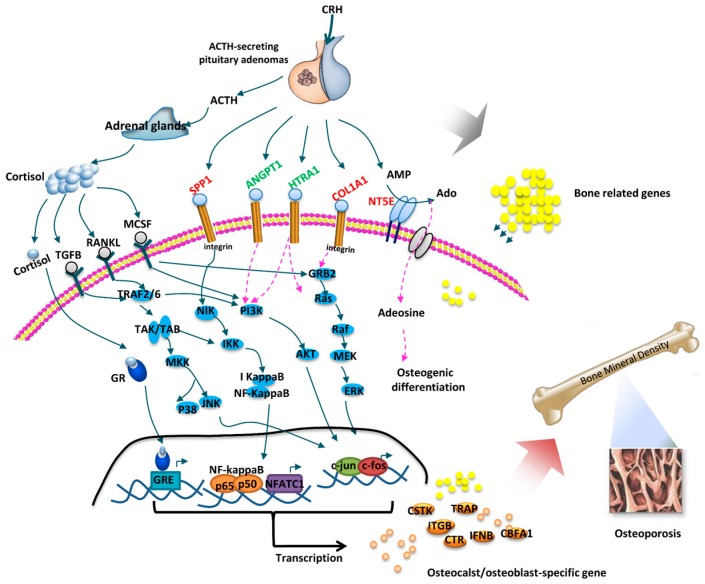
Potential molecular mechanisms of osteoporosis in ACTH-secreting pituitary adenomas. A summary schematic shows the potential signalling pathways for osteoporosis induced by ACTH-secreting pituitary adenomas. It is mediated by three actions: (1) excessive cortisol exerts direct inhibitory effect on osteoblasts or deregulates RANKL (Receptor Activator for Nuclear Factor-κB Ligand), MCSF (Macrophage Colony Stimulating Factor) and TGF β (Transforming Growth Factor-β) pathways; (2) *SPP1*, *COL1A1*, *NT5E*, *HTRA1* and *ANGPT1* genes secrete from the pituitary to target the skeletal system through well-known bone related pathways; (3) other bone related genes are also involved in this process. Up-regulated genes are coloured in red font; down-regulated genes are coloured in green font. Unverified genes are black. Solid line is the signal transduction that has been confirmed, dotted line is the signal transduction that requires further confirmation. These pathways refer to the literature mining and pathway databases. CRH, Corticotropin-Releasing Hormone; ACTH, Adrenocorticotropic Hormone; GR, glucocorticoid receptors; GRE, glucocorticoid-responsive elements.

*SPP1*, *COL1A1*, *NT5E*, *HTRA1* and *ANGPT1* genes all encoded five secreted proteins according to the Secreting Protein Database (Available online: http://spd.cbi.pku.edu.cn/). Elevated SPP1, COL1A1 and NT5E proteins may be secreted from the pituitary tumor to directly target the skeletal system, with resultant osteoporosis, while downregulated HTRA1 and ANGPT1 may deregulate in bone formation and development. Endogenous cortisol excess has a profound effect on bone metabolism, acting at many aspects. On one hand, cortisol combines with glucocorticoid receptors (GR) to exert an inhibitory effect on osteoblasts [26]. On the other hand, cortisol can increase the expression of RANKL (receptor activator of NF-κB ligand, which enhances osteoclastogenesis) and enhance the expression of the macrophage colony-stimulating factor (MCSF). Previous studies have shown that the RANKL (Receptor Activator for Nuclear Factor-κB Ligand), MCSF (Macrophage Colony Stimulating Factor) and TGF β (Transforming Growth Factor-β) pathways played critical roles in osteoporosis (Figure 5) [15,27,28,29]. Therefore, we further elucidated whether these five secreted protein signalling pathways were implicated in bone fractures of CD. Using literature mining and pathway databases, we found that these five secreted protein signalling pathways were associated with well-known bone related pathways (Figure 5): SPP1, also known as osteopontin, signalled through integrin receptors, activating the NIK-IKK-NF-κB pathway [30,31]. COL1A1 triggered the integrin receptors, enhancing the GRB2-RAS-RAF-MEK-ERK pathway [32]. NT5E catalysed AMP (adenosine monophosphate) to Ado, inducing adenosine signalling, and influencing osteogenic differentiation [33,34]. ANGPT1 and HTRA1 deregulated the PI3K-AKT pathway [35,36] (Figure 5). These pathways have established molecular links from the pituitary tumors to osteoporosis development, they may act directly or indirectly to impair osteo-blastogenic differentiation, decrease osteoblast function and increase osteoblastic apoptosis, and thus may explain a molecular mechanism for CD pathogenesis. SPP1 signalling and COL1A1 signalling in osteoporosis were previously reported [30,31,37]; and NT5E, HTRA1 and ANGPT1 signalling pathways reported here require further confirmation. Our results suggest that 138 genes related to BMD Z scores may also play roles in ACTH-secreting pituitary adenoma related to bone fracture (Figure 5).

## 3. Discussion

In this report, we analysed the clinical characteristics and whole transcriptomic changes from a large cohort of CD patients. Osteoporosis is a major worldwide health problem. Although it is often underestimated in CD patients [38,39], osteoporosis was distinguished from 106 clinical characteristics analysed here.

Interestingly, most clinical characteristics of the metaboic syndrome and heart disease, commonly observed in CD patients, were not highly associated with tumor size.

Previous studies have shown that glucocorticoid receptor mutation or gene deficiency can cause glucocorticoid resistance [18,40,41,42,43], resulting in increased secretion of cortisol. Cortisol is crucial for the regulation of bone metabolism at many levels [15], however exact mechanisms are not fully understood due to a lack of human cell lines and animal models, as well as difficulty in obtaining large cohort of CD patients.

This investigation of a rare osteoporosis of a pituitary origin constitutes a strategy for elucidating its molecular pathogenesis. We found a cluster of genes potentially involved in this type of osteoporosis of CD patients. Importantly, we discovered five genes encoding secreted proteins, signalling through bone formation and development pathways involved in the osteoporosis of CD patients. These five deregulated secreted proteins might, in part, be secreted from the pituitary tumor to their skeletal targets or partly through inducing glucocorticoid resistance to lead to osteoporosis. SPP1 (osteopontin) is a highly phosphorylated sialoprotein that is a prominent component of the mineralized extracellular matrices of bones. In the process of bone resorption, osteopontin interacts with integrin and has a role in the adherence of osteoclasts to the bone surface [44]. COL1A1 is a fibril-forming collagen found in most connective tissues and is abundant in bone. Studies showed that mutations in this gene were associated with osteogenesis imperfecta and idiopathic osteoporosis [37]. Although the molecluar mechanism of NT5E, HTRA1 and ANGPT1 in osteoporosis has not been studied yet, the signaling pathways regulated by them are closely related to the signal pathways of bone development and formation. So, the occurrence and development of osteoporosis in CD is a complex regulation process, which is not only related to high cortisol secretion, but also related to the precise regulation of a series of bone related genes.

Thus, our analysis of clinical characteristics in 118 CD patients revealed that pituitary tumor size and ACTH-related hormones were correlated with BMD. Our genotype analysis confirmed that the tumor, hormones and bone metabolism were enriched in whole transcriptomic changes. The phenotype-genotype association analysis revealed a cluster of genes related to BMD in CD patients. We selected five genes encoding secreted proteins to perform experiments in tumor tissues and the ANPT of CD patients and further analysed their signalling transduction pathways to elucidate a molecular mechanism underlying osteoporosis in CD patients.

## 4. Materials and Methods

### 4.1. Patients and Specimens

The protocol was approved by the Ethical Committee of Peking Union Medical College Hospital (045-2011, 1 September 2011, Beijing, China). Informed consent was obtained from each patient before surgery. The methods were carried out in accordance with the approved guidelines. We obtained 118 pathologically proven fresh ACTH-secreting pituitary adenoma specimens for this study from the Department of Neurosurgery, Peking Union Medical College Hospital (Beijing, China) between 16 May 2012 and 25 July 2013. A total of 38 paired ACTH-secreting pituitary adenoma specimens and adjacent non-tumorous pituitary tissues (ANPT) were obtained from 38 female patients; independently, 52 ACTH-secreting pituitary adenoma specimens and 11 ANPT were collected from female patients. We also acquired 13 independent ACTH-secreting pituitary adenomas and four ANPT samples from male patients.

To achieve postoperative endocrine remission, selective adenomectomy was performed with complete removal of the microadenoma and appropriate resection of the limited surrounding area, as reported [45,46] and previously performed by us [47]. Exclusion criteria of the ANPT group patients included (1) aged ≤19 years; (2) Fertility requirement; (3) Pituitary tumor surrounded by pseudocapsule, thus the tumor could be completely removed along the pseudocapsule with no residual normal surrounding tissue [48] and (4) macroadenoma, remnant/normal tissue ≤1/3 of the whole pituitary after surgery. Inclusion criteria of the ANPT group patients included (1) married, with child/children, no further fertility requirement; (2) Severe signs and symptoms of Cushing’s disease; (3) microadenoma, tumor size on MRI ≤5 mm and (4) normal pituitary function at preoperative assessment.

Tissue fragments were immediately frozen in liquid nitrogen under ribonuclease-free conditions at the time of surgery and stored at 80 °C until protein/RNA isolation was performed. All samples were pathologically reassessed by two pathologists, and the percentage of tumor cells was 80% or more in all ACTH-secreting pituitary adenoma specimens; the normal tissues were not contaminated by tumor cells. None of the patients had received radiotherapy or chemotherapy before resection.

### 4.2. Bone Mineral Density of Bone Metabolism

Bone mineral density was measured in the lumbar spine (L1–4) in the anteroposterior (AP) projection and in the right femur using Dual energy X-ray absorptiometry (DEXA). We defined osteopenia as a BMD Z score for the lumbar AP or the right femur of −1 to −2.5 SD (Standard Deviation); we defined osteoporosis as a BMD for the lumbar AP or the right femur when the score was lower than −2.5 SD [49].

### 4.3. RNA-Seq Data Sets

We randomly selected nine paired pituitary tumors and ANPT samples from 38 paired samples to establish gene expression profiles with RNA-seq. The total RNA was isolated using TRIzol reagent (Invitrogen, Carlsbad, CA, USA) according to the manufacturer’s instructions. An RNA deep-sequencing assay was analysed by the BerryGenomics Corporation of China using the Illumina HiSeq™ 2000 (Towne Centre Drive, San Diego, CA, USA). Details of the experiment were as follows: expected library size: 300 bp; read length: 100 nt; and sequencing strategy: paired-end sequencing.

### 4.4. Mapping Reads to the Human Genome and Transcriptome

We downloaded genome and transcriptome reference sequences from the UCSC (University of California Santa Cruz) website [50] (version hg19, Available online: http://genome.ucsc.edu/). Clean reads were aligned to the reference genome and transcriptome using TopHat [51]. TopHat internally used the fast-aligner Bowtie and performed an ab initio identification of splice junctions without relying on gene annotation. For splice junction detection, we set the parameters to allow intron sizes between 40 bp and 1 Mb. The anchor size was set at 8 bp, and no more than one mismatch was allowed in the anchor region. We removed the threshold on the minimum isoform frequency so that the splice junctions belonging to rare isoforms would also be reported. The multi-alignment, which was greater than 40, was discarded to prevent interference.

### 4.5. Estimating Expression Abundance and Normalisation

Reads that could be uniquely mapped to a gene were used to calculate the expression level. A total of 32,186 genes, with names beginning with “NM“, were chosen as the protein-coding genes [24]. The gene expression level was measured by standard FPKM (fragments per kilobase of exons per million mappable reads) based on the final read assignments described in the previous section [23]. A total of 31,933 genes expressed (FPKM > 0), while 21,508 genes had high expression levels with FPKM > 1.

### 4.6. Differentially Expressed Gene Analysis

The differential index (DI) was introduced to identify differentially expressed genes among the nine paired samples on the following processes:

Firstly, fold change of each pair of samples was normalised to a 0–1 value; 1 was the most significant difference, and 0 was no difference between the two paired samples.

Secondly, the differential index (*DI*) was the average of nine normalised 0–1 values.

The formula was defined as below:

(1) $$DI=(\pm )\frac{\sum_{i=1}^{n}1-\frac{1}{\mathrm{fold}}}{n}$$

in which “fold” was the original value of the genes fold change and “*n*” was the number of samples. If gene expression was up-regulated in tumor groups compared with ANPT groups, the *DI* was affixed with “+”; otherwise, the *DI* was affixed with “−”.

Adjust fold change (*FC*) = 1/(1 − |*DI*|).

We identified the differentially expressed genes between the ACTH-secreting pituitary adenomas and adjacent non-tumorous pituitary tissues based on the following criteria: (1) *DI* > 0.6 (which corresponds to *FC* > 2.5) and (2) the differentially expressed gene covers at least six patients. Finally, 423 genes were selected for subsequent analyses, including 153 up-regulated genes and 270 down-regulated genes.

### 4.7. Gene Functional Enrichment Analysis

The 423 differentially expressed genes were functionally clustered using the online gene-annotation enrichment tool David (Available online: http://david.abcc.ncifcrf.gov/) [25]; the GO terms of the top ten functional clusters were filtered based on the *p* value <0.05 and FDR < 0.1 and further described by Cytoscape software.

### 4.8. Quantitative RT–PCR

The total RNA was isolated using Trizol reagent in accordance with the manufacturer’s protocol (Invitrogen). The quality and quantity of RNA were assessed using NanoDrop 2000 (Thermo Scientific, Waltham, MA, USA). cDNA was synthesised with 1 microgram of total RNA using a High-Capacity cDNA Reverse Transcription Kit (Applied Biosystems, Foster City, CA, USA). qRT-PCR was performed with LightCycler 480 SYBR Green Master (Roche, Basel, Switzerland) according to the manufacturer’s instructions. Signals were detected using the Roche LC480 Real-Time PCR System (Roche, Switzerland). The relative expression level was determined using the ΔΔ*C*_t_ method and normalised to the GAPDH levels. All primer sequences for qRT-PCR are listed in Table S15.

### 4.9. Protein Isolation and Western Blot

To explore the protein expression in ACTH-secreting pituitary adenomas, proteins were precipitated from phenol-ethanol supernatant after RNA isolation according to the manufacturer’s protocol. Equal amounts (20 µg) of protein were separated using a SDS-polyacrylamide gel, transferred onto nitrocellulose membrane (NC) and incubated in blocking solution (TBS buffer containing 5% non-fat dry milk) for 1 h at room temperature. The membranes were then incubated overnight at 4 °C with primary antibody (anti-SPP1, epitomic #2671-1, 1:500; anti-COL1A1, epitomic, #6690-1, 1:500; anti-NT5E, epitomic, #5362-1, 1:1000; anti-HTRA1, Abcam, ab38611, 1:200; anti-ANGPT1, Abcam, ab8451,1:200; anti-GAPDH:1:2000, epitomic, #5632-1) and subsequently with HRP-conjugated secondary antibody (Santa Cruz Biotechnology, Santa Cruz, CA, USA) for 1 h at room temperature. Immunocomplexes were visualised using the ECL (electrochemiluminescence) Western Blotting Detection Reagents (Millipore, ‎Billerica, MA, USA) and detected via a Kodak film exposure detection system. Quantification of the bands was performed using the Quantity-One software (Bio-Rad, ‎Hercules, CA, USA).

### 4.10. Statistical Analysis

We used a one sample Wilcoxon signed rank test to determine the differences of clinical parameters between the pituitary tumor patients and healthy controls. The normal reference values were used as estimations of the population mean. We used the Mann-Whitney *U* test in measurement data and the chi-squared test in enumeration data to identify the differences in clinical parameters between two different groups of pituitary tumor patients. We used Spearman’s rank correlation coefficient analysis among nine or 109 patients’ pheno-phenotypes for correlation calculations and Pearson’s coefficient correlation for nine patients’ pheno-genotypes for a correlative study. The Benjamini-Hochberg method was used to control for the false discovery rate (FDR) for multiple testing corrections. A paired-sample Wilcoxon signed rank test was used to identify differences in mRNA and protein expression levels between paired samples of pituitary tumor patients, while the Mann-Whitney *U* test was used to identify gene expression levels between two unpaired groups. The Wilcoxon signed rank test, Mann-Whitney *U* test and correlation statistical analyses were performed with SPSS16·0 for Windows (SPSS, Inc., Chicago, IL, USA). Multiple testing correction FDR values were calculated using Software R script. *p* Values of less than 0.05, derived from a two-tailed test of all analyses, were considered statistically significant.

## 5. Conclusions

In this study, we use the Next Generation Sequencing technology to profile the gene expressions of ACTH-secreting pituitary adenomas. Our findings provide new insight into the potential mechanisms of the development of osteoporosis due to ACTH-secreting pituitary adenomas, thus opening new potential avenues to intervene with this disease. Further evaluation of our discoveries is warranted.

## Figures and Tables

**Table 1:** Clinical characteristics of 118 Adrenocorticotrophin (ACTH)-secreting pituitary adenomas patients.

Clinical Characteristics	Training Set (*n* = 9)	Validation Set (*n* = 109)	*p* Value
Age mean (SD)-years	34.0 (9.5)	35.6 (12.6)	0.82
Sex, female No. (%)	9 (100%)	92 (84.4%)	0.35
Symptoms and Signs No. (%)			
Pain in back and loin	4 (44.4%)	38 (34.9%)	0.72
Osteoporosis	4 (44.4%)	59 (54.1%)	0.73
Pathological fracture	2 (22.2%)	31 (28.4%)	1.00
Coexisting Conditions No. (%)			
Hypertension	9 (100%)	90 (82.6%)	0.35
Diabetes	0 (0%)	42 (38.5%)	0.03
Hyperlipidemia	4 (44.4%)	51 (46.8%)	0.99
Hormone level mean (SD)			
8 AM plasma ACTH-pg/mL	120.7 (91.3)	85.1 (59.2)	0.06
8 AM total serum cortisol-µg/dL	34.6 (16.7)	34.4 (11.5)	0.95
Midnight total serum cortisol-µg/dL	27.8 (11.0)	25.7 (9.0) ^a^	0.80
24 h urinary free cortisol-µg/24 h	417.1 (421.2)	576.5 (559.9)	0.24
Bone Metabolism Mean (SD)			
Ca-mmol/L	2.3 (0.1)	2.3 (0.1)	0.25
P-mmol/L	1.2 (0.2)	1.1 (0.2)	0.21
ALP-U/L	70.3 (18.4)	93.4 (69.6)	0.34
PTH-pg/mL	48.1 (42.4)	62.7 (44.5)	0.14
MRI tumor maximum diameter Mean (SD)-mm	5.3 (1.5)	8.4 (7.2)	0.25
Bone Mineral Density Z-scores (Median (IQR))	*n* = 7 ^b^	*n* = 68 ^c^	
Lumbar vertebra 1	−0.8 (2.5)	−1.5 (1.3)	0.47
Lumbar vertebra 2	−1.7 (2.0)	−1.5 (1.4)	0.77
Lumbar vertebra 3	−1.3 (1.2)	−1.3 (1.0)	0.95
Lumbar vertebra 4	−1.5 (1.6)	−1.3 (1.0)	0.50
Lumbar vertebra 1–2	−1.0 (2.3)	−1.5 (1.4)	0.73
Lumbar vertebra 1–4	−1.3 (1.8)	−1.5 (1.1)	0.91
Lumbar vertebra 2–4	−1.5 (1.5)	−1.4 (1.4)	0.70
Neck of femur	−1.3 (1.2)	−1.2 (0.8)	0.52
Greater trochanter	−1.2 (1.2)	−1.0 (1.0)	0.60
Total haunch bone	−1.1 (1.3)	−0.9 (1.0)	0.47

^a^ Midnight total serum cortisol and 24 h urinary free cortisol were detected in 83 patients; ^b,c^ Seven patients in the training set measured bone mineral density (BMD) with Dual energy X-rays absorptiometry (DEXA) at lumbar spines and right femurs, while in validation set, 68 patients measured BMD. Mann-Whitney tests were used in measurement data and chi-square tests were used in enumeration data. SD, Standard Deviation; ALP, alkaline phosphatase; PTH, parathyroid hormone; MRI, magnetic resonance imaging; IQR, interquartile range.

## Supplementary Materials

Supplementary materials can be found at www.mdpi.com/1422-0067/17/10/1654/s1.
