# Supplementary material for: Phenotype-Genotype Association Analysis of ACTH-Secreting Pituitary Adenoma and Its Molecular Link to Patient Osteoporosis
Source: Int J Mol Sci. 2016 Sep 29;17(10):1654. doi: 10.3390/ijms17101654 (PMC5085687; doi:10.3390/ijms17101654)
Supplement: Supplementary file 1 [file ijms-17-01654-s001.pdf]

Supplementary Material: Phenotype-Genotype Association Analysis of ACTH-Secreting Pituitary Adenoma and Its Molecular Link to Patient Osteoporosis

Renzhi Wang, Yakun Yang, Miaomiao Sheng, Dechao Bu, Fengming Huang, Xiaohai Liu, Cuiqi Zhou, Congxin Dai, Bowen Sun, Jindong Zhu, Yi Qiao, Yong Yao, Huijuan Zhu, Lin Lu, Hui Pan, Ming Feng, Kan Deng, Bing Xing, Wei Lian, Yi Zhao and Chengyu Jiang

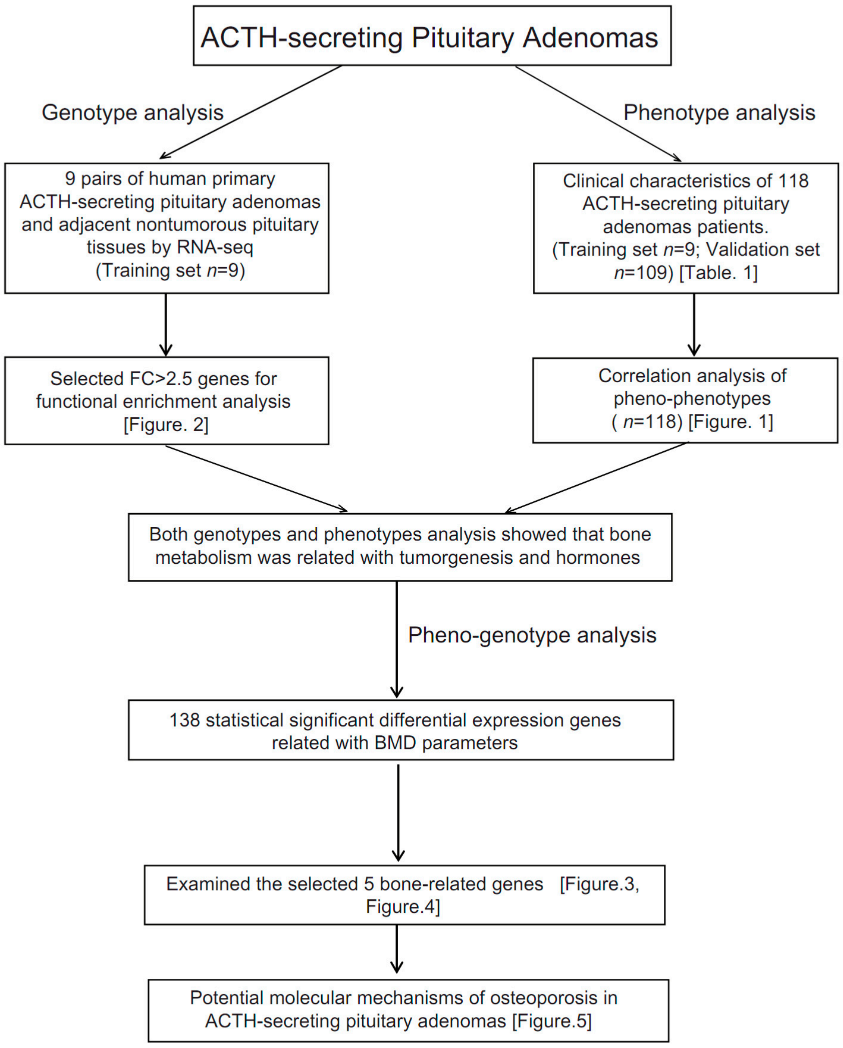

Figure S1. A framework of ACTH-secreting pituitary adenoma pheno-genotype analysis.

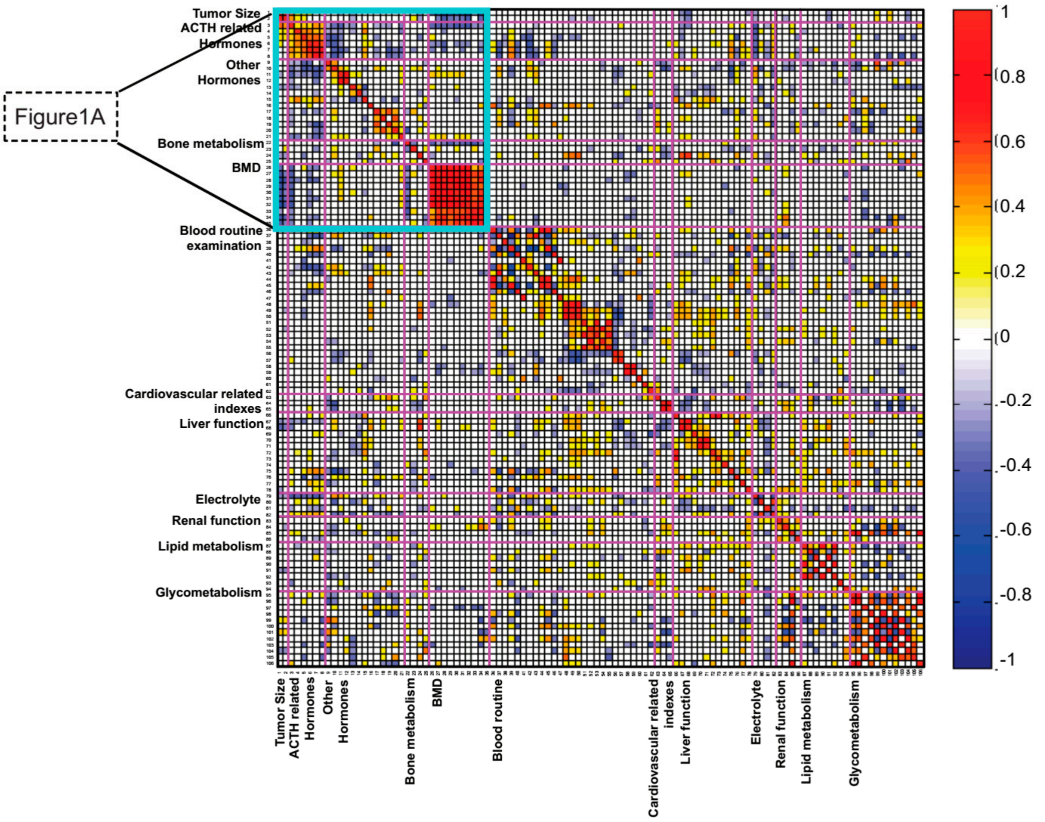

Figure S2. Spearman's rank correlation coefficient analysis of 106 clinical characteristics indices. A total of 106 clinical characteristics indices were processed for SRCC analysis. These phenotypes were shown in Table S1. The heat map of the SRCC results (*p* value < 0.05, FDR < 0.05) was generated using Matlab 7.0 software. Warm colours represent positive correlations, and cold colours represent negative correlations. White indicates no association.

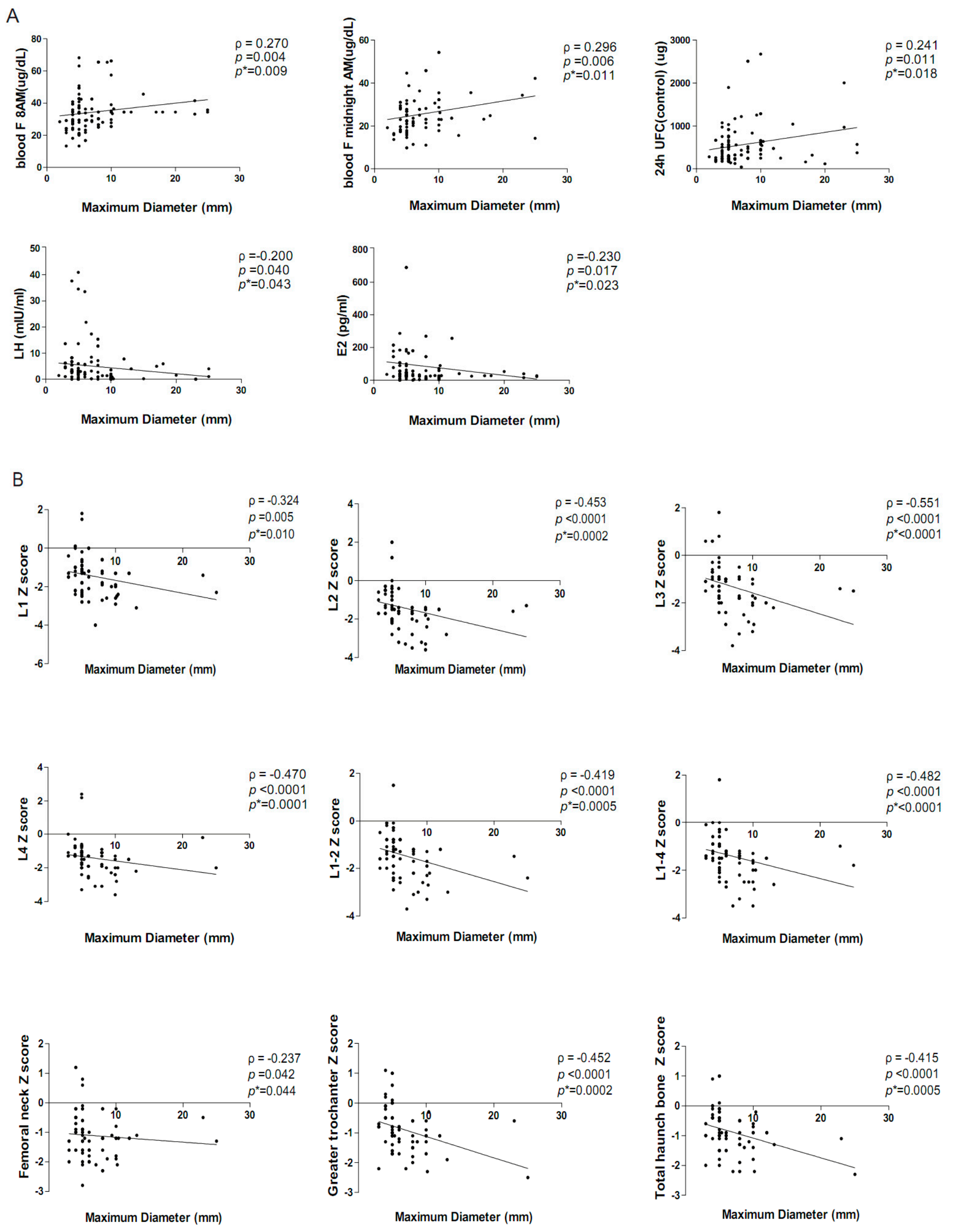

Figure S3. The maximum tumor diameter correlated with ACTH-secreting pituitary adenoma-related hormones, LH, E2 and age-matched bone mineral density (BMD) indices in ACTH-secreting pituitary adenomas. (**A**) The maximum tumor diameter was positively correlated with ACTH-secreting Pituitary Adenoma Related Hormones: 8 AM cortisol, midnight AM cortisol, 24 h UFC and was negatively correlated with LH and E2 levels; (**B**) The maximum tumor diameter negatively correlated with the age-matched BMD indices: L1, L2, L3, L4, L1-2, L1-4, the neck of the femur, the greater trochanter and the total haunch bone. Spearman's rank correlation analysis (Rho), *p* value and the Benjamini & Hochberg multiple testing correction (*p* *) were used to control for false discovery rate (FDR) and were provided in each graph.

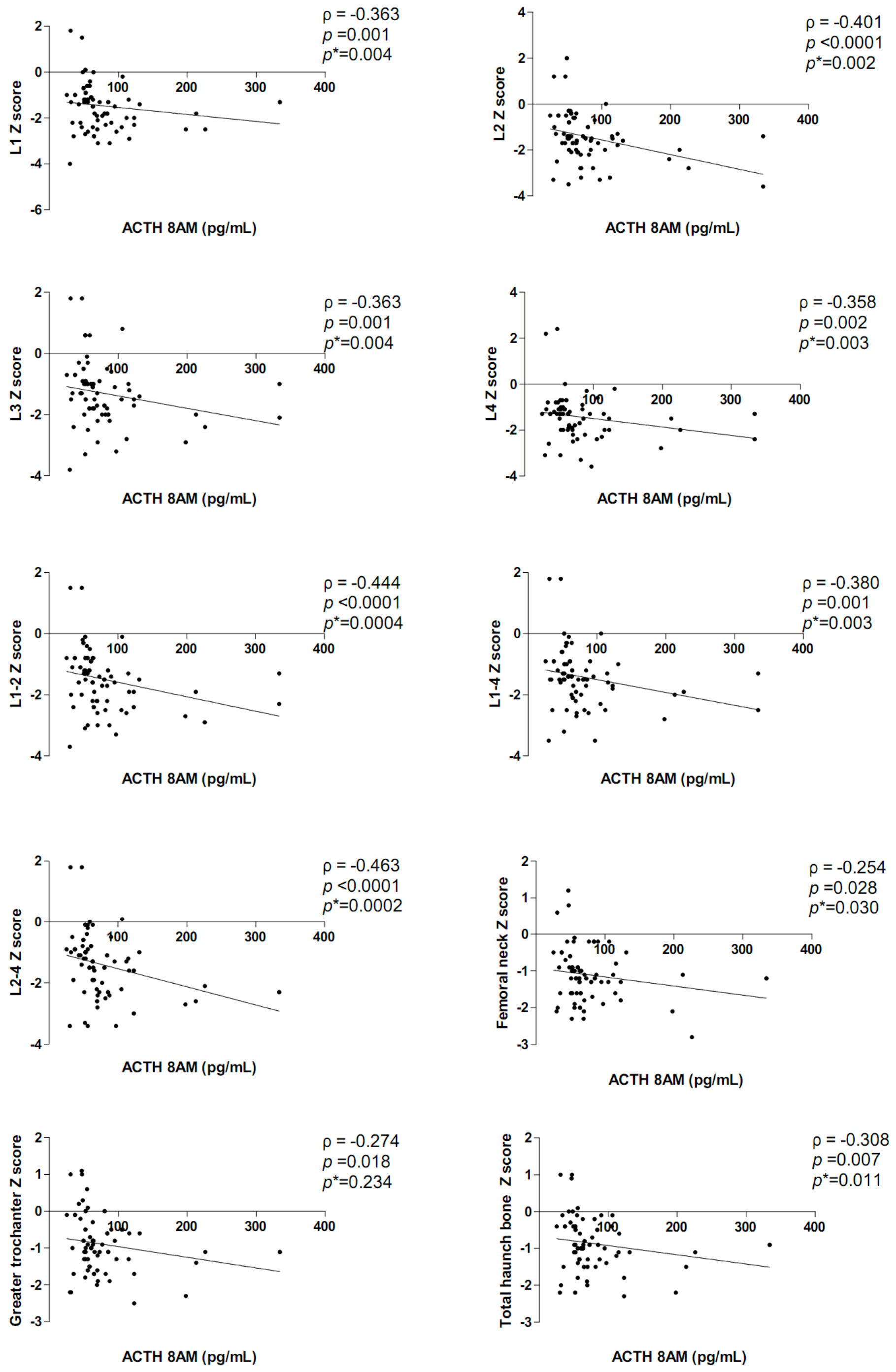

Figure S4. The 8 AM plasma ACTH Levels was inversely correlated with age-matched bone mineral density (BMD) indices in ACTH-secreting pituitary adenoma patients. The 8 AM plasma ACTH was negatively correlated with the age-matched BMD indices: L1, L2, L3, L4, L1-2, L1-4, L2-4, the neck of the femur, the greater trochanter and the total haunch bone. Spearman's rank correlation analysis (Rho), *p* value and the Benjamini & Hochberg multiple testing correction (*p* *) were used to control for false discovery rate (FDR) and were provided in each graph.

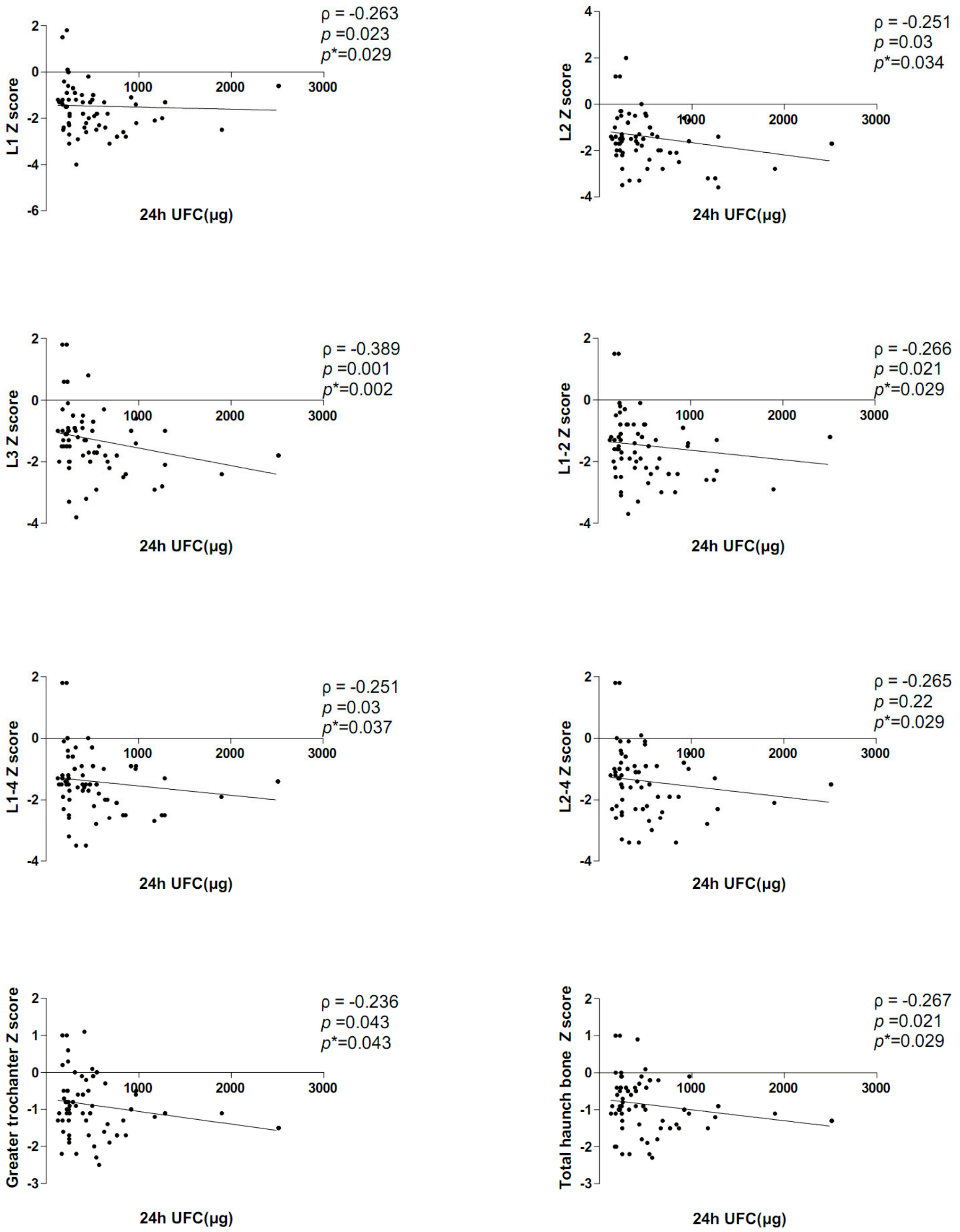

Figure S5. 24 h urinary-free cortisol level was inversely correlated with age-matched bone mineral density (BMD) indices in ACTH-secreting pituitary adenoma patients. The 24-h urinary-free cortisol level was correlated inversely with the age-matched BMD indices: L1, L2, L3, L1-2, L1-4, the greater trochanter and the total haunch bone. Spearman's rank correlation analysis (Rho), the *p* value and the Benjamini & Hochberg multiple testing correction (*p* *) were used to control for false discovery rate (FDR) and were provided in each graph.

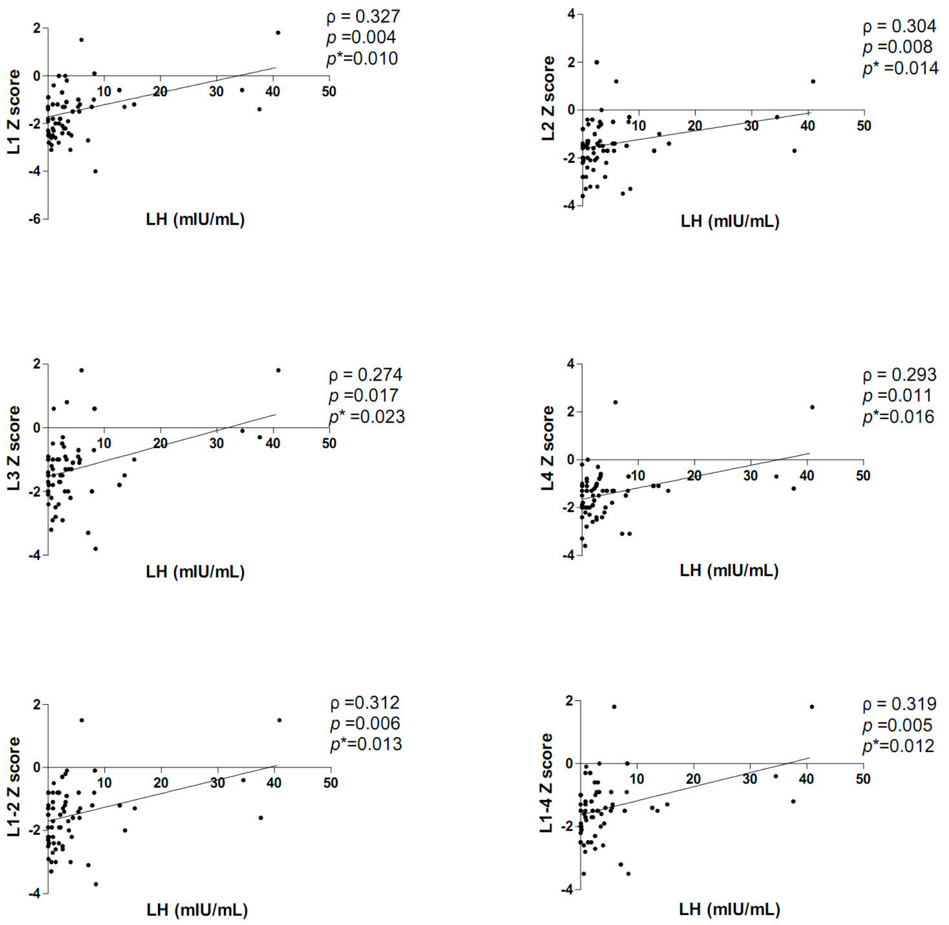

Figure S6. The LH level was positively correlated with age-matched bone mineral density (BMD) indices in ACTH-secreting pituitary adenoma patients. LH level was positively correlated with the age-matched BMD indices: L1, L2, L3, L4, L1-2 and L1-4. Spearman's rank correlation analysis (Rho), the *p* value and the Benjamini & Hochberg multiple testing correction (*p* *) were used to control for false discovery rate (FDR) and were provided in each graph.

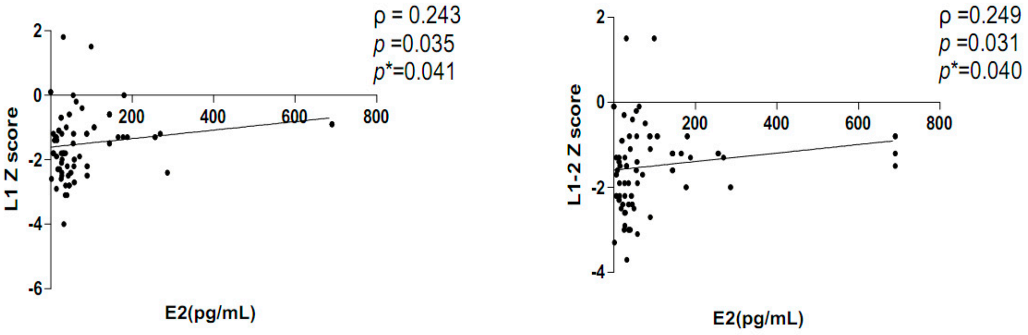

Figure S7. The estradiol level was positively correlated with the age-matched bone mineral density (BMD) indices in ACTH-secreting pituitary adenoma patients. Estradiol level was positively correlated with the age-matched BMD indices: L1 and L1-2. Spearman's rank correlation analysis (Rho), the *p* value and the Benjamini & Hochberg multiple testing correction (*p* *) were used to control for false discovery rate (FDR) and were provided in each graph.

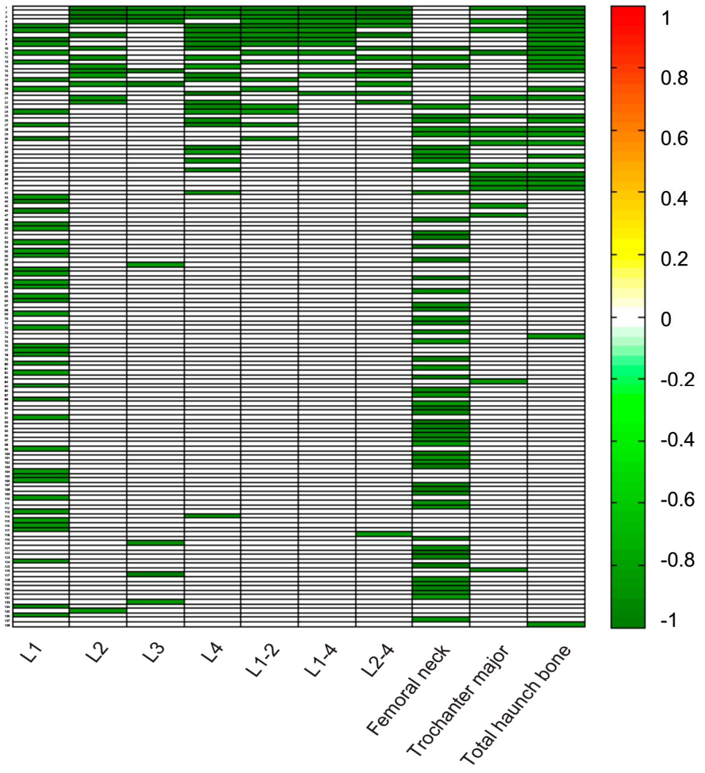

Figure S8. Spearman's Rank Correlation Coefficient (SRCC) analysis of age-matched bone mineral density (BMD) phenotypes and genotypes.The 10 clinical age-matched BMD phenotypes and 423 differentially expressed encoding genes in the ACTH-secreting pituitary adenomas were processed for SRCC analysis. There were 138 genes that were highly related with BMD indices (Tables S8 and S9). The heat map of the SRCC results (p value < 0.05, FDR < 0.05) was generated using Matlab 7.0 software. Warm colours represent positive correlations, cold colours represent negative correlations and white indicates no association.

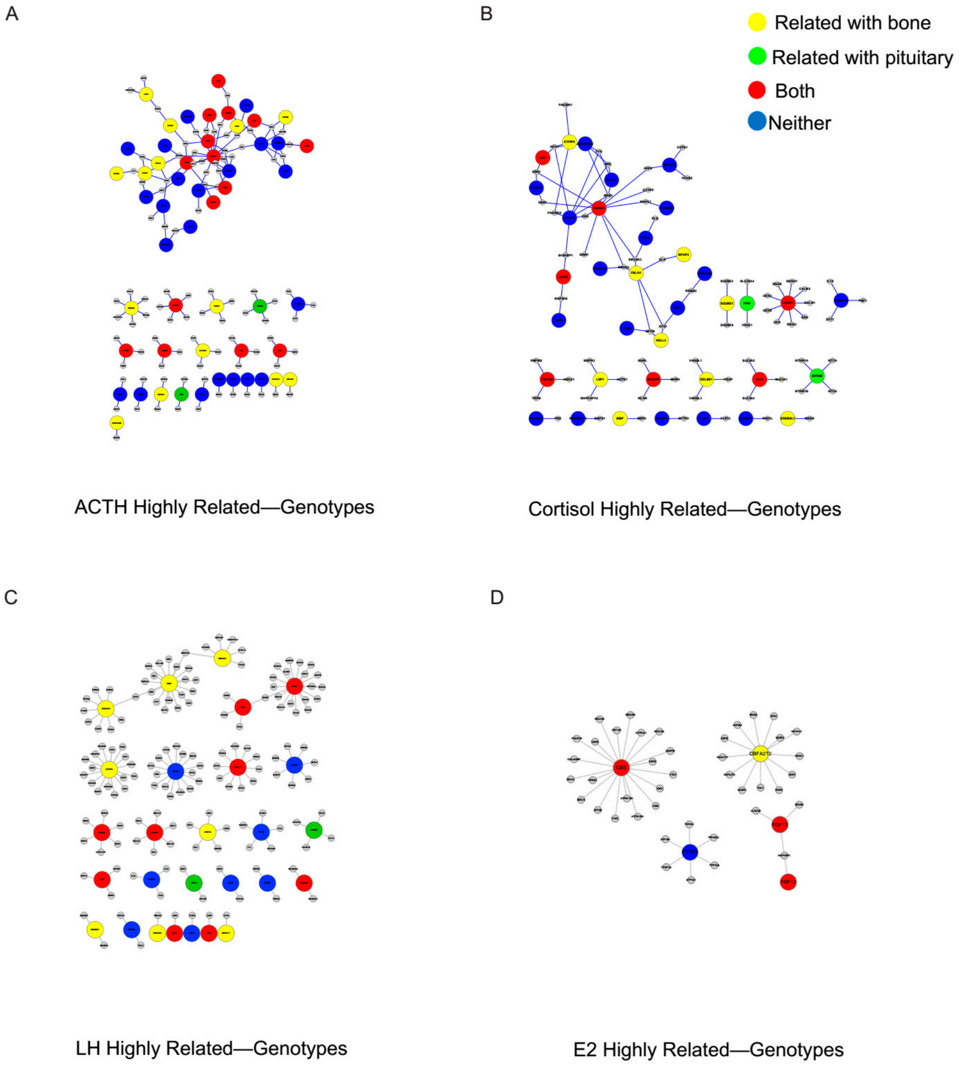

Figure S9. Gene expression profile of ACTH-related hormones in the PPI connection. Literature mining of the genes highly related to ACTH (**A**), genes highly related to cortisol (**B**), genes related to LH (**C**) and genes related to E2 (**D**) assessed for PPI connection. Red represents genes reported for bone and pituitary adenoma fields. Yellow and green represent genes reported for the bone or pituitary adenomas field, respectively. Blue represents genes not reported for bone or pituitary adenomas.

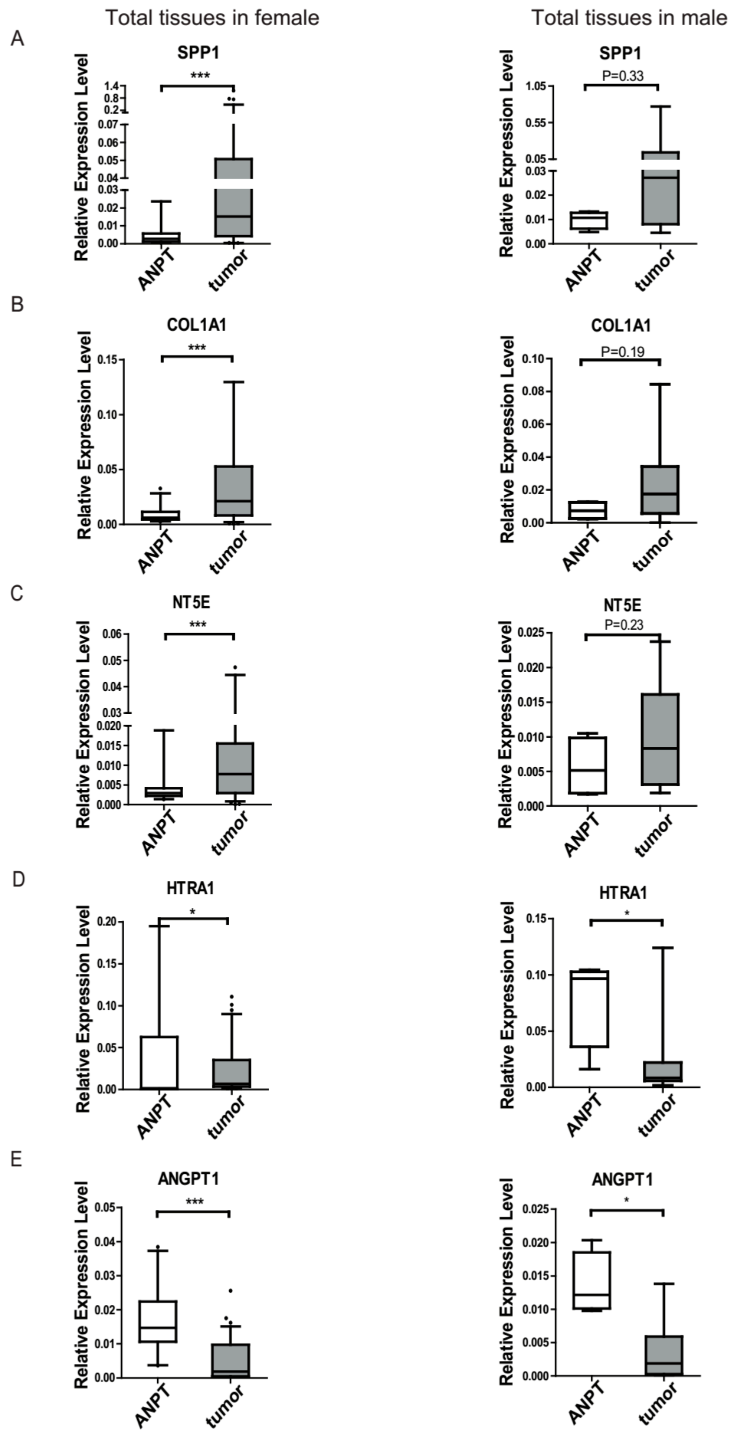

Figure S10. Signature profile of BMD Z score-related gene expression in ACTH-secreting pituitary adenomas. Comparison of expression levels of *SPP1* (**A**), *COL1A1* (**B**), *NT5E* (**C**), *HTRA1* (**D**) and *ANGPT1* (**E**) between ANPT and ACTH-secreting pituitary adenomas via qRT-PCR, including 92 female tissues (29 female paired tissues and 63 female unpaired tissues) and 17 male tissues (ANPT, $n = 4$; tumor, $n = 13$). The data are representative of three technical repeats with the median (quartiles). (Mann-Whitney test, p * < 0.05, p *** < 0.001. ANPT versus tumors).

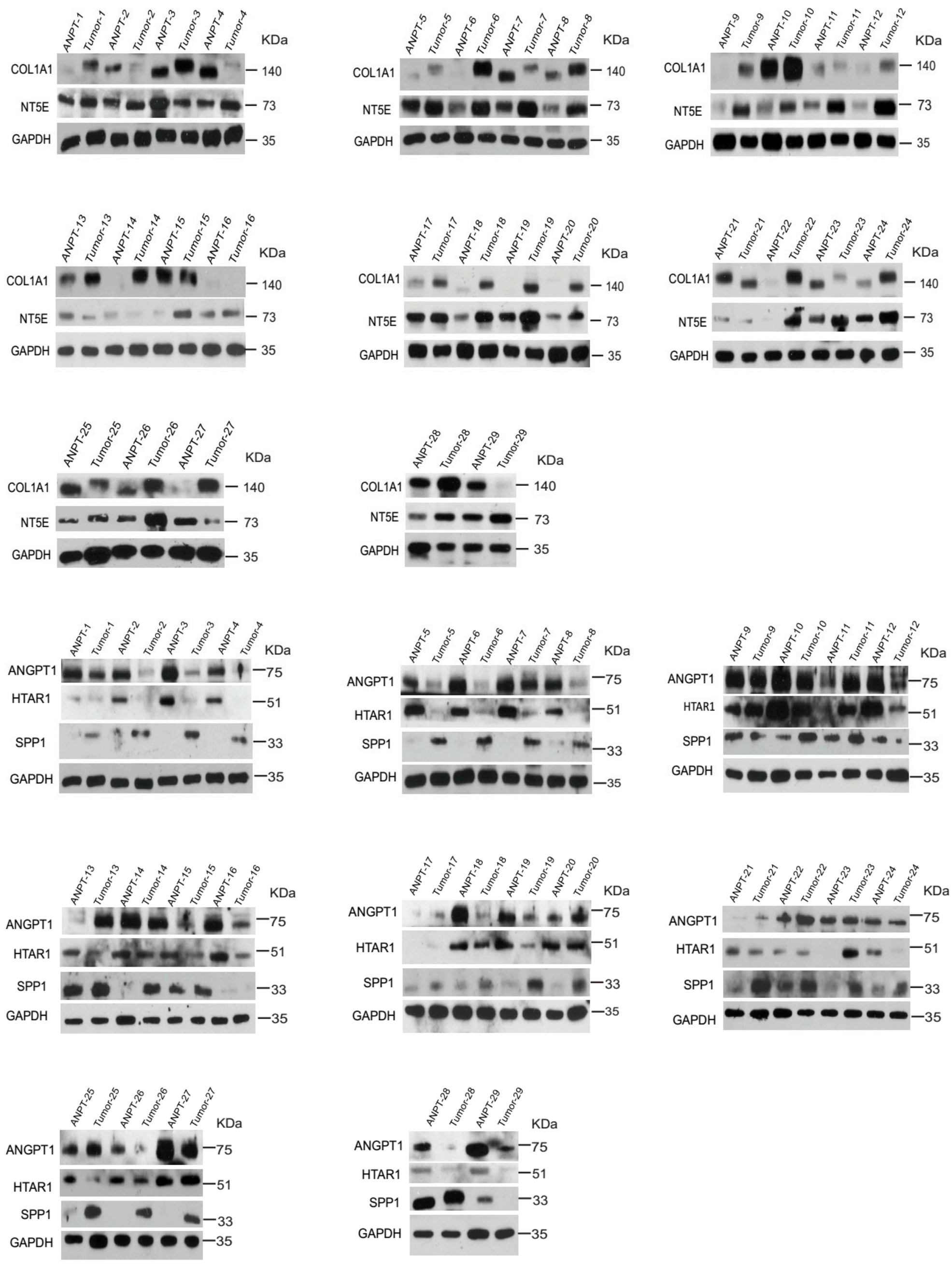

Figure S11. Full-length blots of Figure 4. Western blot assessedthe expression levels of SPP1, COL1A1, NT5E, HTRA1 and ANGPT1 protein between 29 female pairs of ANPT and ACTH-secreting pituitary adenomas.

Table S1. Spearman results of pheno-phenotypes.

ID	Phenotypes	0.8–1	0.5–0.8	(−0.5)–0.5	(−0.8)–(−0.5)	(−1)–(−0.8)	Total
	Tumor size						
1	Maximum diameter	2	1	22	2	0	27
2	Gross tumor volume	2	0	24	0	0	26
	ACTH-secreting pituitary adenoma related hormones						
3	ACTH 8AM	1	1	39	0	0	41
4	8AM F	1	0	23	0	0	24
5	0AM F	1	1	31	0	0	33
6	24 h UFC (control)	1	2	38	0	0	41
7	24 h UFC (low-dose DST)	1	3	37	0	0	41
8	24 h UFC (high-dose DST)	1	2	35	0	0	38
	Other pituitary related hormones						
9	GH	1	1	30	0	0	32
10	IGF-1	1	0	44	1	0	46
11	LH	1	1	31	0	0	33
12	FSH	1	1	14	0	0	16
13	E2	1	0	22	0	0	23
14	PRL	1	0	16	0	0	17
15	TSTO	1	0	33	0	0	34
16	PROG	1	1	29	0	0	31
17	FT3	1	1	29	0	0	31
18	FT4	1	1	23	0	0	25
19	T3	1	1	25	0	0	27
20	T4	1	1	20	0	0	22
21	TSH	1	0	25	0	0	26
	Bone metabolism						
22	PTH	1	0	17	0	0	18
23	P	1	0	25	0	0	26
24	Ca	1	3	28	0	0	32
25	ALP	1	0	25	0	0	26
	Age-matched bone mineral density (BMD)						
26	L1	3	4	19	0	0	26
27	L2	5	5	16	0	0	26
28	L3	4	6	11	1	0	22
29	L4	3	7	9	0	0	19
30	L1-2	5	5	14	0	0	24
31	L1-4	7	3	13	0	0	23
32	L2-4	6	4	7	1	0	18
33	Neck of femur	1	8	9	0	0	18
34	Greater trochanter	2	7	21	0	0	30
35	Total haunch bone	1	7	11	0	0	19
	Routine blood chemistry						
36	WAC	2	2	27	0	0	31
37	LY%	1	2	37	1	1	42
38	MONO%	0	0	16	0	0	16
39	NEUT%	1	1	41	3	1	47
40	EOS	2	0	31	2	0	35
41	BASO	1	0	12	0	0	13
42	LUC	1	1	28	2	0	32
43	LY#	1	1	33	1	0	36
44	MONO#	1	4	28	0	0	33
45	NEUT#	2	2	27	3	0	34
46	EOS#	2	0	29	0	0	31
47	BASO#	2	0	19	0	0	21
48	RBC	1	3	29	0	0	33
49	HGB	2	2	35	0	0	39
50	HCT	2	3	30	1	0	36
51	MCV	3	0	16	0	0	19
52	MCHC	2	1	28	0	0	31
53	MCH	3	1	20	0	0	24
54	CHCM	2	0	23	0	0	25
55	CH	3	0	16	0	0	19

Table S1. *Cont.*

ID	Phenotypes	0.8–1	0.5–0.8	(−0.5)–0.5	(−0.8)–(−0.5)	(−1)–(−0.8)	Total
56	RDW-C	1	1	30	0	0	32
57	HDW	1	1	21	1	0	24
58	PLT	1	1	18	0	0	20
59	PCT	1	1	23	0	0	25
60	PDW	1	0	16	0	0	17
61	MPV	1	0	13	0	0	14
62	ESR	1	0	26	0	0	27
	Cardiovascular related indexes						
63	hsCRP	1	0	31	0	0	32
64	SBP	1	1	22	0	0	24
65	DBP	1	1	24	0	0	26
	Liver function						
66	ALT	1	2	25	0	0	28
67	TP	2	1	37	0	0	40
68	Alb	2	1	36	0	0	39
69	A/G	1	0	28	0	0	29
70	Tbil	2	0	29	0	0	31
71	Dbil	2	0	28	0	0	30
72	GGT	1	1	40	0	0	42
73	AST	1	1	16	0	0	18
74	TBA	1	0	19	0	0	20
75	LD	1	0	42	1	0	44
76	ChE	1	0	32	0	0	33
77	ADA	1	0	32	0	0	33
78	PA	1	0	40	0	0	41
	Electrolyte						
79	K	1	0	37	0	0	38
80	Na	1	1	29	0	0	31
81	Cl	1	0	43	0	0	44
82	TCO_2	1	1	36	0	0	38
	Renal function						
83	Cr(E)	1	0	20	0	0	21
84	Urea	1	0	30	2	0	33
85	Glu	3	4	40	0	0	47
86	UA	1	0	16	0	0	17
	Lipid metabolism						
87	TC	3	0	30	0	0	33
88	TG	1	1	24	0	0	26
89	HDL-C	2	0	29	0	0	31
90	LDL-C	3	0	17	0	0	20
91	ApoA1	2	0	20	0	0	22
92	ApoB	3	1	22	0	0	26
93	Lp(a)	1	0	15	0	0	16
94	FFA	1	0	27	0	0	28
	Glycometabolism						
95	blood gloucose	2	3	40	0	0	45
96	GLU 0 min	2	5	17	0	0	24
97	INS 0 min	1	2	25	0	0	28
98	GLU 30 min	1	6	21	0	0	28
99	INS 30 min	1	3	28	1	0	33
100	GLU 60 min	1	5	27	0	0	33
101	INS 60 min	1	2	23	2	0	28
102	GLU 120 min	1	5	31	1	0	38
103	INS 120 min	1	4	21	0	0	26
104	GLU 180 min	1	3	25	0	0	29
105	INS 180 min	1	4	14	0	0	19
106	HbAlc	1	4	19	0	0	24

Table S2. Maximum diameter highly related-clinical indexes.

Clinical Indexes	*n*	Spearman	*p* Value	FDR
Gross tumor volume	110	0.941	<0.001	<0.001
ACTH 8 AM	110	0.501	<0.001	<0.001
0 AM F	84	0.296	0.006	0.011
8 AM F	110	0.270	0.004	0.009
24 h UFC (control)	110	0.241	0.011	0.018
LH	107	−0.199	0.040	0.043
Ca	109	−0.221	0.021	0.027
E2	107	−0.230	0.017	0.023
Neck of femur	75	−0.237	0.042	0.044
L1	75	−0.324	0.005	0.010
Total haunch bone	75	−0.415	<0.001	0.001
L1-2	75	−0.419	<0.001	0.001
Greater trochanter	75	−0.452	<0.001	<0.001
L2	75	−0.453	<0.001	<0.001
L4	75	−0.470	<0.001	<0.001
L1-4	75	−0.482	<0.001	<0.001
L3	75	−0.551	<0.001	<0.001
L2-4	75	−0.581	<0.001	<0.001

Table S3. ACTH 8AM highly related-osteo indexes.

Osteo Indexes	*n*	Spearman	*p* Value	FDR
Neck of femur	75	−0.254	0.028	0.030
Greater trochanter	75	−0.274	0.018	0.023
Total haunch bone	75	−0.308	0.007	0.011
L4	75	−0.358	0.002	0.003
L1	75	−0.363	0.001	0.004
L3	75	−0.363	0.001	0.004
L1-4	75	−0.379	0.001	0.003
L2	75	−0.401	<0.001	0.002
L1-2	75	−0.444	<0.001	<0.001
L2-4	75	−0.463	<0.001	<0.001

Table S4. 24 h UFC (control) highly related-osteo.

Osteo Indexes	*n*	Spearman	*p* Value	FDR
Greater trochanter	75	−0.236	0.043	0.043
L2	75	−0.251	0.030	0.034
L1-4	75	−0.251	0.030	0.037
L1	75	−0.263	0.023	0.029
L2-4	75	−0.265	0.022	0.029
L1-2	75	−0.266	0.021	0.029
Total haunch bone	75	−0.267	0.021	0.029
L3	75	−0.389	0.001	0.002

Table S5. LH highly related-osteo indexes.

Osteo Indexes	*n*	Spearman	*p* Value	FDR
L1	75	0.327	0.004	<0.001
L1-4	75	0.319	0.005	<0.001
L1-2	75	0.312	0.006	0.001
L2	75	0.304	0.008	0.010
L4	75	0.293	0.011	0.011
L3	75	0.274	0.017	0.013

Table S6. E2 highly related-osteo indexes.

Osteo Indexes	*n*	Spearman	*p* Value	FDR
L1	75	0.243	0.035	0.041
L1-2	75	0.249	0.031	0.040

Table S7. Information of sequencing quality control.

ID	Tissue	Library Size (bp)	Strand Specific	Sequencing Type	Library Size (bp)	Read Length (nt)	Total Reads	Total Basepairs	Reads Mapped to Genome	Mapping Ratio
N1	tumor	300	NO	Pair-end	300	100	73,614,480	7,361,448,000	60,612,808	82.34%
	ANPTs	300	NO	Pair-end	300	100	78,173,560	7,817,356,000	62,375,857	79.79%
N2	tumor	300	NO	Pair-end	300	100	78,173,560	7,817,356,000	56,070,088	71.73%
	ANPTs	300	NO	Pair-end	300	100	89,010,912	8,901,091,200	73,630,521	82.72%
N3	tumor	300	NO	Pair-end	300	100	79,580,300	7,958,030,000	64,790,623	81.42%
	ANPTs	300	NO	Pair-end	300	100	76,619,924	7,661,992,400	64,033,275	83.57%
N4	tumor	300	NO	Pair-end	300	100	76,996,314	7,699,631,400	62,752,759	81.50%
	ANPTs	300	NO	Pair-end	300	100	90,919,768	9,091,976,800	46,035,736	50.63%
N5	tumor	300	YES	Pair-end	300	100	124,465,980	12,446,598,000	101,145,421	81.26%
	ANPTs	300	YES	Pair-end	300	100	64,252,872	6,425,287,200	52,554,201	81.79%
N6	tumor	300	YES	Pair-end	300	100	48,795,594	4,879,559,400	41,181,643	84.40%
	ANPTs	300	YES	Pair-end	300	100	47,704,480	4,770,448,000	40,219,630	84.31%
N7	tumor	300	YES	Pair-end	300	100	68,686,500	6,868,650,000	57,919,244	84.32%
	ANPTs	300	YES	Pair-end	300	100	75,849,422	7,584,942,200	63,327,495	83.49%
N8	tumor	300	YES	Pair-end	300	100	90,908,180	9,090,818,000	75,853,891	83.44%
	ANPTs	300	YES	Pair-end	300	100	87,935,006	8,793,500,600	53,538,621	60.88%
N9	tumor	300	YES	Pair-end	300	100	73,968,232	7,396,823,200	62,944,377	85.10%
	ANPTs	300	YES	Pair-end	300	100	78,229,188	7,822,918,800	63,552,258	81.24%

Table S8. Age-matched bone mineral density (BMD) highly related-genotypes.

Gene	Count of Highly Related BMD	FC	L1	L2	L3	L4	L1–2	L1–4	L2–4	Neck of Femur	Greater Trochanter	Total Haunch Bone
COL1A1	8	3.01	—	0.93	0.89	0.86	0.88	0.93	0.96	—	0.82	0.91
RAB3B	7	4.79	—	0.98	0.85	0.87	0.92	0.96	0.97	—	—	0.97
INSR	7	3.36	—	0.98	0.91	0.84	0.84	0.91	0.98	—	—	0.89
RFX4	7	3.13	—	0.94	0.92	—	0.82	0.85	0.90	—	0.82	0.98
LYPD1	6	3.32	0.87	—	—	0.97	0.92	0.89	0.83	—	—	0.86
PITX1	6	2.88	0.85	—	—	0.92	0.90	0.87	—	—	0.82	0.90
CNGA4	6	2.57	—	0.95	—	0.93	0.89	0.94	0.97	—	—	0.91
STK17B	5	3.46	0.98	—	—	0.85	0.91	0.84	—	—	—	0.82
NT5E	5	3.00	0.86	—	—	0.92	0.90	0.86	—	—	—	0.84
PPAPDC1A	5	2.82	—	0.86	—	1.00	—	—	0.91	0.99	—	0.99
ACOT7	5	2.66	0.86	—	—	—	0.89	0.86	—	—	0.95	0.90
GADD45B	5	2.60	—	0.90	—	0.86	—	—	0.87	0.86	—	0.83
ATP2B2	4	3.41	0.84	—	—	—	0.87	0.81	—	—	—	0.81
BAMBI	4	3.08	—	0.85	—	0.84	—	—	—	0.92	—	0.87
SIX2	4	2.79	—	0.87	0.93	—	—	—	0.82	—	—	0.84
CYFIP2	4	2.69	—	0.82	—	0.96	—	0.81	0.86	—	—	—
MYH6	3	5.97	0.88	—	—	0.85	0.83	—	—	—	—	—
KIAA1024	3	5.94	—	0.85	0.94	—	—	—	0.85	—	—	—
HFM1	3	4.70	0.84	—	—	—	0.86	—	—	—	—	0.89
RASEF	3	4.52	—	—	—	0.94	—	0.81	0.83	—	—	—
ZBTB49	3	3.17	—	0.86	—	—	—	—	—	—	0.84	0.90
C1orf95	3	2.97	—	0.86	—	0.83	—	—	0.90	—	—	—
IGFBP3	3	2.78	—	—	—	0.93	0.81	—	—	0.87	—	—
ATP6V1C2	3	2.68	0.90	—	—	0.85	0.84	—	—	—	—	—
PDE7A	3	2.68	—	—	—	—	—	—	—	0.87	0.83	0.84
OLFM2	3	2.66	—	—	—	0.89	—	—	—	0.90	—	0.81
PITPNM2	3	2.59	0.83	—	—	0.88	0.81	—	—	—	—	—
CHGA	3	2.57	—	—	—	—	—	—	—	0.87	0.86	0.90
TCF7L2	3	2.53	—	—	—	—	—	—	—	0.87	0.81	0.83
HIGD1B	2	6.64	0.93	—	—	—	0.84	—	—	—	—	—
FABP7	2	3.50	—	—	—	—	—	—	—	—	0.85	0.84
LHX3	2	3.45	—	—	—	0.88	—	—	—	0.87	—	—
COL11A2	2	3.23	—	—	—	0.84	—	—	—	0.87	—	—
PI16	2	3.10	—	—	—	—	—	—	—	0.86	—	0.83

Table S8. *Cont.*

Gene	Count of Highly Related BMD	FC	L1	L2	L3	L4	L1–2	L1–4	L2–4	Neck of Femur	Greater Trochanter	Total Haunch Bone
SOCS2	2	3.00	—	—	—	0.85	—	—	—	0.83	—	—
MYCL1	2	2.99	—	—	—	—	—	—	—	—	0.83	0.83
SLC38A3	2	2.96	—	—	—	0.85	—	—	—	0.86	—	—
SCD5	2	2.93	—	—	—	—	—	—	—	—	0.88	0.91
THBD	2	2.88	—	—	—	—	—	—	—	—	0.90	0.89
KIAA1211	2	2.82	—	—	—	—	—	—	—	—	0.83	0.87
PPARGC1A	2	2.82	—	—	—	—	—	—	—	—	0.90	0.92
PPIF	2	2.58	—	—	—	0.84	—	—	—	0.87	—	—
CRHR1	1	7.47	0.83	—	—	—	—	—	—	—	—	—
CHRNE	1	5.16	0.88	—	—	—	—	—	—	—	—	—
DPP6	1	4.99	—	—	—	—	—	—	—	—	0.85	—
TMEM100	1	4.90	0.93	—	—	—	—	—	—	—	—	—
FOXL2	1	4.79	—	—	—	—	—	—	—	—	0.82	—
MSX1	1	4.50	—	—	—	—	—	—	—	0.94	—	—
NELL2	1	4.45	0.83	—	—	—	—	—	—	—	—	—
PTGDR2	1	4.26	0.83	—	—	—	—	—	—	—	—	—
C14orf180	1	4.20	—	—	—	—	—	—	—	0.89	—	—
GPR149	1	4.17	—	—	—	—	—	—	—	0.97	—	—
MEIS2	1	4.16	0.83	—	—	—	—	—	—	—	—	—
CACNG2	1	4.11	—	—	—	—	—	—	—	0.95	—	—
GPC3	1	4.11	0.83	—	—	—	—	—	—	—	—	—
HSD11B1	1	4.00	0.83	—	—	—	—	—	—	—	—	—
NTNG1	1	3.92	—	—	—	—	—	—	—	0.96	—	—
TRIM2	1	3.77	—	—	0.82	—	—	—	—	—	—	—
GNAL	1	3.77	0.82	—	—	—	—	—	—	—	—	—
TLE3	1	3.75	0.83	—	—	—	—	—	—	—	—	—
ALDH1A2	1	3.74	—	—	—	—	—	—	—	0.92	—	—
MYT1L	1	3.74	0.82	—	—	—	—	—	—	—	—	—
SALL1	1	3.73	0.83	—	—	—	—	—	—	—	—	—
FMOD	1	3.71	—	—	—	—	—	—	—	0.90	—	—
S100A13	1	3.69	0.83	—	—	—	—	—	—	—	—	—
RSPO3	1	3.61	0.85	—	—	—	—	—	—	—	—	—
TSPAN12	1	3.54	—	—	—	—	—	—	—	0.89	—	—
TSPAN8	1	3.52	—	—	—	—	—	—	—	0.88	—	—

Table S8. *Cont.*

Gene	Count of Highly Related BMD	FC	L1	L2	L3	L4	L1–2	L1–4	L2–4	Neck of Femur	Greater Trochanter	Total Haunch Bone
SCGN	1	3.46	0.82	—	—	—	—	—	—	—	—	—
S100A1	1	3.42	—	—	—	—	—	—	—	0.85	—	—
KCNH8	1	3.39	—	—	—	—	—	—	—	0.87	—	—
PLEKHG5	1	3.37	0.83	—	—	—	—	—	—	—	—	—
ARMC4	1	3.36	—	—	—	—	—	—	—	0.82	—	—
HTRA1	1	3.35	—	—	—	—	—	—	—	—	—	0.82
PLCD3	1	3.33	—	—	—	—	—	—	—	0.87	—	—
FAM163A	1	3.28	0.81	—	—	—	—	—	—	—	—	—
PPP1R13L	1	3.26	0.83	—	—	—	—	—	—	—	—	—
ANGPT1	1	3.25	0.83	—	—	—	—	—	—	—	—	—
VSTM1	1	3.25	—	—	—	—	—	—	—	0.96	—	—
GNRHR	1	3.21	0.83	—	—	—	—	—	—	—	—	—
ZNF521	1	3.20	—	—	—	—	—	—	—	0.85	—	—
PITX2	1	3.11	0.83	—	—	—	—	—	—	—	—	—
SLC16A2	1	3.10	—	—	—	—	—	—	—	0.93	—	—
ESR1	1	3.09	—	—	—	—	—	—	—	—	0.82	—
USH1C	1	3.08	0.82	—	—	—	—	—	—	—	—	—
IGFBP5	1	3.08	—	—	—	—	—	—	—	0.91	—	—
PCP4	1	3.07	—	—	—	—	—	—	—	0.85	—	—
ATG9B	1	3.06	0.91	—	—	—	—	—	—	—	—	—
SCN1B	1	3.06	—	—	—	—	—	—	—	0.82	—	—
DHRS3	1	3.05	—	—	—	—	—	—	—	0.95	—	—
CPVL	1	3.03	—	—	—	—	—	—	—	0.91	—	—
COL4A6	1	3.01	0.83	—	—	—	—	—	—	—	—	—
MATN2	1	3.00	—	—	—	—	—	—	—	0.96	—	—
LGR5	1	2.98	—	—	—	—	—	—	—	0.95	—	—
F3	1	2.97	—	—	—	—	—	—	—	0.86	—	—
APOL4	1	2.97	—	—	—	—	—	—	—	0.92	—	—
RNASE1	1	2.95	—	—	—	—	—	—	—	0.88	—	—
C19orf33	1	2.95	—	—	—	—	—	—	—	0.84	—	—
NEUROD4	1	2.95	0.83	—	—	—	—	—	—	—	—	—
GPX3	1	2.94	—	—	—	—	—	—	—	0.91	—	—
C7	1	2.92	—	—	—	—	—	—	—	0.82	—	—
CPNE4	1	2.88	—	—	—	—	—	—	—	0.84	—	—

Table S8. *Cont.*

Gene	Count of Highly Related BMD	FC	L1	L2	L3	L4	L1–2	L1–4	L2–4	Neck of Femur	Greater Trochanter	Total Haunch Bone
PHGDH	1	2.88	—	—	—	—	—	—	—	0.98	—	—
AMPD3	1	2.86	0.83	—	—	—	—	—	—	—	—	—
PLTP	1	2.86	0.82	—	—	—	—	—	—	—	—	—
SPP1	1	2.86	0.83	—	—	—	—	—	—	—	—	—
PKD1L1	1	2.85	—	—	—	—	—	—	—	0.90	—	—
AMOT	1	2.85	—	—	—	—	—	—	—	0.98	—	—
CYS1	1	2.83	—	—	—	—	—	—	—	0.86	—	—
C17orf72	1	2.83	0.83	—	—	—	—	—	—	—	—	—
AGR3	1	2.83	—	—	—	—	—	—	—	0.87	—	—
FLRT1	1	2.83	—	—	—	—	—	—	—	0.84	—	—
ZNF516	1	2.81	0.84	—	—	—	—	—	—	—	—	—
UCK2	1	2.81	—	—	—	0.83	—	—	—	—	—	—
IGSF1	1	2.80	0.83	—	—	—	—	—	—	—	—	—
FZD7	1	2.79	0.87	—	—	—	—	—	—	—	—	—
FGF1	1	2.77	0.83	—	—	—	—	—	—	—	—	—
MFAP2	1	2.77	—	—	—	—	—	—	0.82	—	—	—
GDPD2	1	2.75	—	—	—	—	—	—	—	0.85	—	—
SYNE2	1	2.70	—	—	0.89	—	—	—	—	—	—	—
ADAM28	1	2.70	—	—	—	—	—	—	—	0.88	—	—
EXOC3L2	1	2.68	—	—	—	—	—	—	—	0.97	—	—
NTRK2	1	2.66	—	—	—	—	—	—	—	0.94	—	—
EPHX1	1	2.66	0.82	—	—	—	—	—	—	—	—	—
FBXO31	1	2.64	—	—	—	—	—	—	—	0.98	—	—
CDH20	1	2.64	—	—	—	—	—	—	—	—	0.81	—
RAB40C	1	2.64	—	—	0.94	—	—	—	—	—	—	—
NFIX	1	2.63	—	—	—	—	—	—	—	0.89	—	—
KIAA1462	1	2.63	—	—	—	—	—	—	—	0.83	—	—
OXT	1	2.61	—	—	—	—	—	—	—	0.91	—	—
CDH22	1	2.60	—	—	—	—	—	—	—	0.85	—	—
SYT12	1	2.60	—	—	—	—	—	—	—	0.84	—	—
PRRG4	1	2.56	—	—	0.84	—	—	—	—	—	—	—
TIMP4	1	2.55	0.84	—	—	—	—	—	—	—	—	—
ADCY2	1	2.54	—	0.83	—	—	—	—	—	—	—	—
GALNT14	1	2.53	0.83	—	—	—	—	—	—	—	—	—
CCL14	1	2.51	—	—	—	—	—	—	—	0.87	—	—
FILIP1	1	2.51	—	—	—	—	—	—	—	—	—	0.85

—: p value > 0.05.

Table S9. Osteo-related genes.

ID	N1		N2		N3		N4		N5		N6		N7		N8		N9		Differentail Recurrence	DI	Avg (Tumor)	Avg (ANPTs)	Gene Symbol	Chr	FC
	Tumor	ANPTs	Tumor	ANPTs	Tumor	ANPTs	Tumor	ANPTs	Tumor	ANPTs	Tumor	ANPTs	Tumor	ANPTs	Tumor	ANPTs	Tumor	ANPTs							
NM_000088	40.10	5.07	43.31	9.33	77.52	7.59	5.41	7.36	16.26	6.24	21.30	3.31	12.44	3.88	24.57	4.79	24.26	5.71	8	0.668	29.4626	5.92063	*COL1A1*	chr17	3.01
NM_002867	78.90	2.25	68.27	2.84	54.60	3.54	2.25	3.76	33.42	1.99	55.90	1.62	16.51	1.43	35.83	1.39	42.35	5.40	8	0.791	43.1134	2.69056	*RAB3B*	chr1	4.79
NM_001079817	88.35	9.62	53.97	9.41	94.81	10.07	7.92	8.51	19.79	6.86	38.24	5.50	10.85	4.89	34.87	5.96	73.20	12.84	8	0.703	46.8891	8.18416	*INSR*	chr19	3.36
NM_213594	0.27	6.65	0.30	4.41	0.36	5.74	3.71	2.80	1.05	3.80	0.00	4.76	4.91	3.03	0.28	5.56	0.00	6.03	7	−0.680	1.20784	4.75336	*RFX4*	chr12	3.13
NM_001077427	1.71	24.28	2.00	16.03	1.94	19.78	11.34	18.63	23.78	58.97	1.51	30.08	33.97	41.80	19.45	36.92	0.58	39.67	6	−0.699	10.6968	31.7958	*LYPD1*	chr2	3.32
NM_002653	21.85	143.56	32.03	158.57	22.98	111.75	101.51	113.15	49.20	100.13	7.88	79.51	47.24	80.58	25.41	81.52	9.77	53.11	7	−0.652	35.3185	102.432	*PITX1*	chr5	2.88
NM_001037329	7.27	0.39	3.94	0.39	4.95	0.19	0.39	0.41	0.26	0.47	2.52	0.16	0.32	0.44	9.48	1.29	1.66	0.17	6	0.612	3.42163	0.436548	*CNGA4*	chr11	2.57
NM_004226	9.69	1.58	7.61	2.07	18.04	1.64	1.77	2.16	7.63	2.11	13.05	1.57	6.68	1.63	6.17	1.71	26.00	3.98	8	0.711	10.7378	2.05126	*STK17B*	chr2	3.46
NM_002526	15.62	1.62	18.26	2.64	13.47	1.33	3.39	1.73	8.57	2.10	31.88	2.07	3.84	5.18	4.71	1.69	17.51	3.64	7	0.667	13.0272	2.44483	*NT5E*	chr6	3.00
NM_001030059	12.02	0.00	7.35	0.08	6.05	0.44	0.05	0.04	1.21	0.05	0.48	0.00	1.70	0.09	0.08	0.00	6.82	0.07	8	0.646	3.97368	0.0855755	*PPAPDC1A*	chr10	2.82
NM_181864	0.84	6.64	1.02	2.80	0.41	3.78	2.36	2.82	3.89	4.71	0.29	2.60	0.55	2.25	0.69	3.52	1.17	5.07	7	−0.624	1.24907	3.80045	*ACOT7*	chr1	2.66
NM_015675	198.82	34.59	169.21	30.85	131.76	32.65	23.16	25.69	138.46	70.46	133.77	23.91	45.20	27.65	184.90	47.83	557.12	110.95	6	0.616	175.822	44.9527	*GADD45B*	chr19	2.60
NM_001001331	9.80	1.39	10.98	2.10	10.23	1.83	1.27	1.48	7.81	1.25	6.86	0.66	5.23	2.17	12.62	2.41	9.43	2.53	8	0.706	8.24858	1.7587	*ATP2B2*	chr3	3.41
NM_012342	1.52	18.37	0.92	11.82	0.44	6.96	6.91	6.29	3.28	6.33	0.48	7.67	2.20	2.59	0.70	6.25	0.04	4.55	6	−0.675	1.83169	7.8706	*BAMBI*	chr10	3.08
NM_016932	3.47	29.58	2.72	11.29	2.20	26.19	38.42	32.44	9.62	25.34	1.12	7.35	4.39	6.23	1.27	10.81	1.38	5.13	7	−0.642	7.1763	17.1509	*SIX2*	chr2	2.79
NM_001037332	18.28	4.45	23.61	3.90	17.16	3.47	4.67	5.18	5.97	2.52	32.45	1.34	10.38	3.42	18.65	3.03	15.00	3.43	8	0.689	16.2413	3.41592	*CYFIP2*	chr5	3.22
NM_002471	49.39	0.60	50.54	0.84	157.14	5.49	0.81	0.77	179.89	5.40	268.46	1.39	29.61	2.19	7.48	2.12	138.85	7.02	8	0.833	98.0186	2.86987	*MYH6*	chr14	5.97
NM_015206	13.75	0.35	6.13	0.32	15.69	0.40	0.18	0.32	8.20	0.60	5.12	0.32	3.30	0.52	9.14	0.40	12.08	0.90	8	0.832	8.17541	0.45839	*KIAA1024*	chr15	5.94
NM_001017975	15.89	0.92	10.66	0.89	17.65	0.89	0.73	0.75	17.69	1.56	42.83	1.23	4.30	1.88	13.73	0.87	26.54	2.74	8	0.787	16.6687	1.30376	*HFM1*	chr1	4.70
NM_152573	23.53	1.49	15.81	1.36	14.93	1.62	1.21	1.87	23.55	4.60	23.17	1.65	27.75	3.07	12.04	1.61	77.62	17.12	8	0.779	24.4024	3.82164	*RASEF*	chr9	4.52
NM_145291	52.97	4.42	57.15	5.20	96.54	5.63	2.14	3.33	9.49	5.04	32.52	2.32	9.07	3.83	38.92	3.33	86.81	11.75	7	0.685	42.8454	4.98444	*ZBTB49*	chr4	3.17
NM_001003665	0.55	4.60	1.01	5.35	1.23	6.07	4.63	4.59	0.99	2.73	0.58	2.60	2.07	3.69	1.26	3.76	0.12	3.82	7	−0.664	1.38323	4.13486	*C1orf95*	chr1	2.97
NM_000598	11.78	122.91	10.50	107.23	4.65	44.56	79.69	70.19	95.49	243.65	17.44	291.84	77.73	90.03	84.99	208.53	21.12	222.18	7	−0.641	44.8201	155.679	*IGFBP3*	chr7	2.78
NM_144583	31.05	2.67	18.26	2.55	50.70	3.55	1.99	2.25	11.61	2.89	17.77	0.67	4.22	2.07	0.00	0.98	28.79	8.09	8	0.627	18.2676	2.85882	*ATP6V1C2*	chr2	2.68
NM_001242318	5.08	1.30	6.05	1.25	5.43	1.19	1.37	1.08	3.28	1.61	4.05	0.59	2.52	1.27	6.39	1.29	6.89	2.30	7	0.627	4.56203	1.31953	*PDE7A*	chr8	2.68
NM_058164	1.63	12.78	1.18	10.43	0.59	12.95	11.70	11.23	2.03	2.74	0.49	7.03	5.26	7.55	1.74	5.72	0.17	6.27	6	−0.624	2.755	8.52246	*OLFM2*	chr19	2.66
NM_020845	21.92	4.85	31.06	7.41	42.19	6.81	5.98	5.87	11.10	5.14	43.14	5.28	13.83	7.21	10.18	6.21	46.83	6.68	6	0.613	25.1373	6.16246	*PITPNM2*	chr12	2.59
NM_001275	89.71	611.70	92.86	670.13	130.97	950.83	400.64	320.79	168.27	325.94	39.17	506.93	383.19	463.42	56.71	392.91	88.14	283.09	6	−0.611	161.074	502.859	*CHGA*	chr14	2.57
NM_001198531	5.88	0.82	7.09	0.75	9.14	0.99	0.51	1.15	1.72	1.27	7.19	0.37	2.32	2.11	2.80	0.41	10.85	0.00	7	0.605	5.27676	0.875456	*TCF7L2*	chr10	2.53
NM_016438	9.98	0.40	11.65	1.13	10.15	5.20	1.77	0.46	38.93	3.73	19.08	0.27	4.02	0.88	10.18	1.05	9.85	0.15	8	0.849	12.8466	1.47395	*HIGD1B*	chr17	6.64
NM_001446	1.39	11.38	1.40	8.69	1.26	23.24	13.41	15.00	4.44	13.54	0.72	17.72	10.59	12.60	0.99	16.92	0.65	8.85	7	−0.714	3.87308	14.2158	*FABP7*	chr6	3.50
NM_014564	1.66	20.07	1.25	15.83	0.90	20.13	15.20	13.21	3.39	11.34	0.00	10.73	6.40	8.51	1.82	8.20	0.00	10.70	7	−0.710	3.40104	13.1909	*LHX3*	chr9	3.45
NM_080681	0.00	3.40	0.00	5.09	0.00	9.38	3.76	2.84	4.64	29.95	0.00	13.49	8.96	5.71	1.59	5.98	0.00	13.69	7	−0.691	2.1043	9.94793	*COL11A2*	chr6	3.23
NM_001199159	1.33	12.95	0.59	6.77	0.24	7.11	5.58	5.48	1.77	3.81	0.18	4.65	2.26	1.70	0.27	1.73	0.06	3.71	7	−0.678	1.36572	5.32201	*PI16*	chr6	3.10
NM_003877	2.75	20.84	1.86	16.28	1.86	15.68	15.90	18.15	7.10	17.16	0.82	15.05	10.68	15.92	10.44	18.19	0.77	17.83	6	−0.667	5.79905	17.2336	*SOCS2*	chr12	3.00
NM_001033082	8.92	1.16	7.58	0.92	14.06	1.43	1.07	1.43	2.71	1.07	8.34	0.31	1.55	1.05	7.36	0.78	23.27	2.77	7	0.665	8.32031	1.21319	*MYCL1*	chr1	2.99
NM_006841	20.59	2.19	21.70	1.60	25.40	1.94	1.91	1.13	1.84	0.67	16.39	0.92	4.10	1.28	0.43	1.06	23.56	1.08	8	0.663	12.8806	1.3204	*SLC38A3*	chr3	2.96
NM_001037582	6.56	42.00	6.95	30.13	5.05	37.69	40.99	39.93	5.42	13.60	1.28	15.28	14.52	18.54	2.07	18.75	2.59	16.91	7	−0.658	9.49237	25.871	*SCD5*	chr4	2.93
NM_000361	70.88	7.48	41.53	6.03	67.34	5.93	5.11	5.24	3.05	3.18	28.90	1.78	3.43	1.84	33.92	2.29	52.58	6.26	6	0.653	34.0825	4.44683	*THBD*	chr20	2.88
NM_020722	3.34	0.60	3.91	0.65	8.09	0.72	0.36	0.36	1.68	0.57	4.96	0.53	1.37	0.73	6.24	0.83	11.06	1.89	7	0.646	4.55588	0.765442	*KIAA1211*	chr4	2.82
NM_013261	3.46	0.71	2.76	0.63	3.43	0.62	0.74	0.92	2.41	1.34	5.69	0.82	2.13	1.11	7.49	1.27	7.25	1.40	6	0.645	3.92915	0.979337	*PPARGC1A*	chr4	2.82
NM_005729	93.19	5.88	124.40	6.18	35.79	5.58	5.43	8.45	15.05	2.25	19.32	1.07	2.91	1.98	3.01	1.74	22.90	1.92	6	0.612	35.7797	3.89561	*PPIF*	chr10	2.58
NM_004382	95.23	0.79	124.65	2.63	53.82	1.02	0.26	0.51	10.72	0.71	13.26	0.00	1.08	0.00	39.62	0.00	18.91	1.70	8	0.866	39.7285	0.816888	*CRHR1*	chr17	7.47
NM_000080	67.86	3.26	70.49	4.61	110.94	5.37	2.07	1.77	54.12	3.08	27.24	0.51	5.06	0.95	7.56	1.45	46.61	5.96	8	0.806	43.5513	2.9954	*CHRNE*	chr17	5.16
NM_001039350	22.08	1.32	27.77	2.70	44.13	1.72	2.53	2.37	5.94	2.03	27.72	1.89	3.80	0.46	14.69	0.79	48.81	1.05	8	0.800	21.9404	1.59285	*DPP6*	chr7	4.99
NM_018286	12.71	1.29	12.28	1.84	17.89	1.84	1.26	1.98	13.33	1.74	27.46	1.73	9.18	1.02	9.84	1.17	70.61	4.14	8	0.796	19.3954	1.86047	*TMEM100*	chr17	4.90
NM_023067	0.38	11.47	0.42	9.54	0.26	12.40	5.75	4.91	1.96	6.56	0.03	9.09	4.77	10.19	0.10	9.62	0.01	7.46	8	−0.791	1.51981	9.02789	*FOXL2*	chr3	4.79

Table S9. *Cont.*

ID	N1		N2		N3		N4		N5		N6		N7		N8		N9		Differentail Recurrence	DI	Avg (Tumor)	Avg (ANPTs)	Gene Symbol	Chr	FC
	Tumor	ANPTs	Tumor	ANPTs	Tumor	ANPTs	Tumor	ANPTs	Tumor	ANPTs	Tumor	ANPTs	Tumor	ANPTs	Tumor	ANPTs	Tumor	ANPTs							
NM_002448	1.69	18.25	0.70	10.44	0.42	8.86	5.81	7.02	2.77	9.25	0.47	7.02	3.26	7.53	0.63	7.12	0.40	5.21	8	−0.778	1.79492	8.96657	*MSX1*	chr4	4.50
NM_001145108	0.28	8.92	0.54	9.19	1.57	13.18	8.96	11.20	1.08	2.40	0.00	5.43	3.06	6.97	0.39	5.62	0.46	8.70	8	−0.775	1.81558	7.9582	*NELL2*	chr12	4.45
NM_004778	3.22	0.41	2.82	0.30	2.90	0.82	0.48	0.23	20.65	2.66	11.64	0.14	3.60	0.81	3.69	0.65	5.06	0.28	9	0.765	6.00721	0.69912	*PTGDR2*	chr11	4.26
NM_001008404	7.11	0.16	31.40	0.22	29.96	0.48	0.15	0.30	26.24	0.70	7.57	0.06	1.74	0.11	0.02	0.07	6.71	0.02	9	0.762	12.3204	0.234088	*C14orf180*	chr14	4.20
NM_001038705	0.24	1.81	0.10	2.43	0.12	1.95	2.46	5.19	1.70	3.72	0.00	4.40	4.97	5.54	0.00	4.06	0.00	2.17	8	−0.760	1.06384	3.4763	*GPR149*	chr3	4.17
NM_170676	0.26	3.64	0.01	3.21	0.00	3.06	3.49	2.82	2.50	6.66	0.00	8.69	2.89	4.12	0.08	6.47	0.00	8.53	7	−0.760	1.02438	5.24318	*MEIS2*	chr15	4.16
NM_006078	4.39	0.85	9.58	0.59	5.34	0.43	0.14	0.39	8.34	1.62	9.24	0.63	2.54	0.83	4.90	0.77	16.42	1.79	9	0.757	6.76393	0.877184	*CACNG2*	chr22	4.11
NM_004484	12.01	36.08	1.26	54.51	2.13	40.00	38.78	35.28	2.87	25.13	0.00	13.86	21.86	48.00	1.35	11.07	0.00	24.15	8	−0.757	8.91792	32.0085	*GPC3*	chrX	4.11
NM_181755	0.83	5.24	0.26	4.03	0.00	4.58	3.84	4.85	6.80	11.67	0.00	13.74	8.99	14.64	0.33	9.23	0.00	16.98	6	−0.750	2.33964	9.4395	*HSD11B1*	chr1	4.00
NM_014917	0.47	5.37	0.08	2.47	0.31	2.62	2.09	2.11	1.22	4.59	0.12	3.07	2.85	5.05	0.82	4.36	0.01	3.75	7	−0.745	0.8861	3.70967	*NTNG1*	chr1	3.92
NM_001130067	1.76	0.00	4.18	0.97	2.31	0.35	0.40	0.62	6.35	0.00	4.59	0.00	4.59	0.00	4.51	0.00	1.24	1.14	7	0.735	3.3256	0.34232	*TRIM2*	chr4	3.77
NM_002071	0.31	3.39	0.00	2.90	0.10	3.13	3.07	3.49	2.63	10.66	0.04	11.62	10.80	14.83	0.35	16.98	0.00	16.78	7	−0.764	1.92189	9.30976	*GNAL*	chr18	4.24
NM_001105192	1.40	4.22	0.81	7.33	0.58	3.36	5.57	7.39	2.18	3.82	0.00	4.54	1.92	4.42	0.08	2.94	0.00	2.53	7	−0.733	1.39317	4.50438	*TLE3*	chr15	3.75
NM_003888	1.78	19.75	0.58	12.23	0.78	12.51	9.46	10.64	3.45	10.03	0.38	13.55	5.73	7.03	0.93	7.31	0.00	16.53	7	−0.733	2.56637	12.176	*ALDH1A2*	chr15	3.74
NM_015025	0.98	6.69	0.42	5.21	0.17	4.28	5.53	5.24	3.45	9.64	0.01	11.01	7.37	9.75	0.29	12.10	0.00	12.14	7	−0.733	2.0259	8.44953	*MYT1L*	chr2	3.74
NM_002968	0.75	7.53	0.42	5.00	0.27	5.05	5.38	4.65	2.89	8.83	0.01	8.69	4.19	6.33	1.44	7.84	0.04	8.87	7	−0.732	1.70952	6.97682	*SALL1*	chr16	3.73
NM_002023	3.01	16.71	1.24	15.73	0.85	15.34	11.97	17.15	2.92	7.77	0.73	6.62	4.14	5.15	0.58	4.94	0.02	4.37	7	−0.731	2.82848	10.4193	*FMOD*	chr1	3.71
NM_005979	0.81	4.35	1.25	6.13	0.33	8.14	0.99	2.95	0.60	2.14	0.00	5.58	0.86	2.87	0.38	4.10	0.74	1.11	8	−0.729	0.663753	4.15109	*S100A13*	chr1	3.69
NM_032784	38.68	1.35	42.10	1.58	70.49	1.70	0.48	1.51	84.49	7.52	242.13	2.32	22.11	2.39	2.32	1.11	256.55	7.53	9	0.723	84.3726	3.00139	*RSPO3*	chr6	3.61
NM_012338	0.69	6.66	0.35	4.96	0.41	6.46	6.91	8.63	5.22	10.97	0.97	8.83	8.07	10.81	1.75	11.22	0.19	9.70	7	−0.717	2.7284	8.69409	*TSPAN12*	chr7	3.54
NM_004616	9.86	1.02	12.36	0.81	15.24	0.58	0.38	0.80	5.16	0.75	22.81	0.79	0.31	0.16	2.59	0.29	16.59	0.92	8	0.716	9.47787	0.679506	*TSPAN8*	chr12	3.52
NM_006998	10.29	71.01	3.98	48.36	1.76	41.69	70.28	82.44	21.44	53.92	0.23	44.79	13.10	20.95	11.04	28.91	2.72	40.21	7	−0.711	14.9832	48.03	*SCGN*	chr6	3.46
NM_006271	10.28	59.33	7.93	83.21	3.98	87.06	21.85	26.85	12.82	20.11	4.41	25.70	4.25	9.26	1.38	5.93	0.00	22.10	7	−0.708	7.43308	37.7261	*S100A1*	chr1	3.42
NM_144633	0.88	7.08	0.17	5.38	0.25	5.33	5.20	7.09	1.94	6.01	0.12	7.16	6.71	7.24	2.70	7.16	0.01	7.87	7	−0.705	1.99743	6.70115	*KCNH8*	chr3	3.39
NM_198681	0.47	3.32	0.49	5.04	0.31	2.89	5.65	4.17	1.10	5.16	0.00	3.74	3.48	4.51	0.05	3.66	0.16	2.48	7	−0.703	1.30106	3.88714	*PLEKHG5*	chr1	3.37
NM_018076	0.45	3.37	0.44	3.73	0.36	2.28	2.73	4.45	2.30	5.03	0.52	10.66	6.57	8.29	2.61	10.14	0.75	7.32	7	−0.702	1.85839	6.14156	*ARMC4*	chr10	3.36
NM_002775	19.77	134.27	11.63	98.22	9.45	70.44	87.43	111.71	38.47	79.78	8.71	108.97	68.77	97.49	10.90	121.24	9.96	68.46	7	−0.702	29.4542	98.9555	*HTRA1*	chr10	3.35
NM_133373	1.26	5.06	0.55	4.10	0.35	3.62	3.47	2.91	1.69	6.27	0.40	1.99	1.07	2.33	0.68	3.14	0.17	2.51	8	−0.700	1.07327	3.54647	*PLCD3*	chr17	3.33
NM_173509	2.99	18.14	1.05	10.89	0.84	17.39	15.60	11.87	9.13	22.70	0.07	26.97	15.84	20.94	0.58	21.58	0.15	43.13	7	−0.696	5.13848	21.5127	*FAM163A*	chr1	3.28
NM_006663	3.16	8.00	2.79	15.23	0.13	3.61	5.13	4.17	1.19	3.07	0.00	7.98	1.73	3.99	0.42	4.79	0.97	4.12	8	−0.694	1.72402	6.10631	*PPP1R13L*	chr19	3.26
NM_001146	0.66	2.88	0.00	2.37	0.01	3.50	2.69	2.01	2.22	6.27	0.00	10.09	5.94	5.73	1.33	7.52	0.02	13.19	7	−0.693	1.43127	5.95102	*ANGPT1*	chr8	3.25
NM_198481	1.04	7.58	0.81	16.20	0.04	3.14	9.66	8.04	0.24	0.32	0.00	6.06	1.89	4.70	0.00	4.27	0.00	3.78	7	−0.692	1.52016	6.00986	*VSTM1*	chr19	3.25
NM_000406	2.13	31.90	1.36	23.03	0.27	3.92	10.05	11.58	0.57	1.45	0.00	13.03	3.26	4.46	0.06	8.26	0.00	10.83	7	−0.689	1.9675	12.0511	*GNRHR*	chr4	3.21
NM_015461	1.57	5.52	0.51	3.89	0.36	4.04	4.14	3.56	1.63	3.98	0.43	4.86	2.93	4.71	0.84	6.71	0.43	6.47	7	−0.687	1.42626	4.86103	*ZNF521*	chr18	3.20
NM_000325	1.02	8.10	0.45	8.99	0.33	9.44	7.77	6.26	0.91	2.06	0.00	4.86	3.51	3.78	0.22	4.32	0.00	3.83	7	−0.678	1.57751	5.73724	*PITX2*	chr4	3.11
NM_006517	5.41	28.10	1.62	19.35	2.02	26.37	20.36	15.83	2.82	6.80	0.47	7.20	6.33	8.99	1.05	9.68	0.27	8.56	7	−0.678	4.4832	14.5416	*SLC16A2*	chrX	3.10
NM_001122741	0.51	4.77	0.39	5.44	0.30	4.17	2.00	1.88	0.73	1.50	0.05	2.20	1.47	2.50	0.23	4.44	0.00	3.63	7	−0.677	0.631294	3.39299	*ESR1*	chr6	3.09
NM_005709	1.31	5.95	1.09	12.32	0.62	8.09	1.73	2.88	4.44	9.37	0.02	13.18	4.66	5.21	1.16	2.63	0.29	17.74	7	−0.676	1.70162	8.5971	*USH1C*	chr11	3.08
NM_000599	32.89	257.26	20.85	240.51	15.81	155.30	162.26	160.05	49.68	138.20	9.79	198.44	121.37	127.03	27.95	140.78	6.21	197.93	7	−0.675	49.6462	179.499	*IGFBP5*	chr2	3.08
NM_006198	6.46	19.65	1.36	21.72	2.54	25.32	13.11	12.07	6.47	17.78	1.32	18.25	4.49	18.08	11.77	18.18	0.32	12.19	7	−0.674	5.31696	18.1396	*PCP4*	chr21	3.07
NM_173681	24.82	2.02	6.45	1.73	12.35	2.08	1.69	2.17	10.29	0.99	21.80	0.52	4.23	0.57	0.61	0.50	5.82	0.95	7	0.674	9.78487	1.28148	*ATG9B*	chr7	3.06
NM_001037	50.63	8.16	78.49	8.23	35.80	7.67	6.04	4.71	18.38	5.18	53.92	4.84	13.04	3.18	5.07	4.11	39.94	2.61	7	0.673	33.4806	5.41018	*SCN1B*	chr19	3.06
NM_004753	6.20	35.05	4.24	39.33	1.88	19.61	22.76	22.77	8.57	27.15	1.92	12.98	9.95	11.91	2.38	10.54	0.67	13.60	7	−0.672	6.50672	21.438	*DHRS3*	chr1	3.05
NM_031311	3.12	7.60	0.50	7.53	1.62	5.39	11.72	13.35	2.51	6.10	0.46	5.35	3.68	6.53	1.51	8.88	0.58	6.89	7	−0.670	2.85666	7.51403	*CPVL*	chr7	3.03
NM_033641	1.97	4.70	0.28	4.23	0.20	3.73	5.54	3.82	2.15	5.49	0.00	8.26	4.50	6.38	0.35	7.51	0.00	5.42	7	−0.667	1.66589	5.50447	*COL4A6*	chrX	3.01
NM_030583	0.64	4.20	0.33	3.93	0.44	3.28	2.18	2.10	2.79	4.18	0.50	4.98	2.94	5.66	0.88	3.55	0.43	4.64	6	−0.667	1.23643	4.05834	*MATN2*	chr8	3.00
NM_003667	22.28	0.05	17.28	0.02	46.26	0.13	0.31	0.04	0.04	0.04	4.85	0.00	0.18	0.23	8.77	0.08	8.30	0.05	7	0.664	12.0301	0.0720564	*LGR5*	chr12	2.98

Table S9. *Cont.*

ID	N1		N2		N3		N4		N5		N6		N7		N8		N9		Differentail Recurrence	DI	Avg (Tumor)	Avg (ANPTs)	Gene Symbol	Chr	FC
	Tumor	ANPTs	Tumor	ANPTs	Tumor	ANPTs	Tumor	ANPTs	Tumor	ANPTs	Tumor	ANPTs	Tumor	ANPTs	Tumor	ANPTs	Tumor	ANPTs							
NM_001993	7.41	43.40	3.49	33.56	3.08	31.12	31.80	34.17	48.25	107.95	3.69	72.62	67.31	78.30	21.46	60.62	1.24	123.24	7	−0.664	20.8595	64.9965	*F3*	chr1	2.97
NM_030643	1.16	8.29	0.35	9.99	0.37	5.91	12.03	21.95	2.28	5.31	0.27	5.83	7.59	10.38	5.93	5.37	0.19	4.55	6	−0.663	3.35235	8.61906	*APOL4*	chr22	2.97
NM_198235	0.77	2.26	0.52	3.38	0.74	4.72	0.65	5.72	0.74	1.57	0.18	1.53	2.25	2.48	0.38	2.36	0.00	1.31	8	−0.661	0.692303	2.81541	*RNASE1*	chr14	2.95
NM_033520	9.42	34.95	5.60	56.88	1.03	33.07	46.23	42.83	17.72	52.77	12.05	66.90	14.97	22.90	3.91	21.95	3.25	14.19	7	−0.661	12.6857	38.4917	*C19orf33*	chr19	2.95
NM_021191	1.38	16.68	0.74	11.69	0.71	5.98	7.25	7.38	6.47	16.08	0.42	35.10	13.88	17.83	16.00	29.80	1.32	22.12	6	−0.661	5.35256	18.0733	*NEUROD4*	chr12	2.95
NM_002084	119.49	590.64	51.79	492.15	39.58	293.41	281.50	274.94	280.70	681.38	60.00	605.99	342.59	377.41	54.43	459.35	31.66	534.36	7	−0.660	140.194	478.847	*GPX3*	chr5	2.94
NM_000587	0.69	3.53	0.19	3.40	0.17	2.52	1.79	1.93	4.23	7.79	0.47	4.19	2.62	2.71	0.24	2.53	0.06	4.78	6	−0.658	1.16174	3.71025	*C7*	chr5	2.92
NM_130808	45.44	3.61	59.26	3.51	65.65	4.84	2.98	3.28	16.67	1.99	30.96	1.79	4.48	2.89	1.99	2.34	58.92	5.10	6	0.653	31.8151	3.25965	*CPNE4*	chr3	2.88
NM_006623	6.38	29.83	3.15	23.93	3.14	21.02	20.40	17.90	13.19	34.59	3.94	27.50	20.46	26.69	6.13	29.96	0.39	22.90	7	−0.652	8.57494	26.034	*PHGDH*	chr1	2.88
NM_001025390	0.17	1.90	0.23	1.84	0.26	2.04	0.95	1.10	0.88	2.02	0.00	1.94	3.24	3.10	1.35	5.37	0.28	2.38	7	−0.651	0.81804	2.41119	*AMPD3*	chr11	2.86
NM_006227	1.85	8.78	0.66	11.28	1.03	9.76	5.52	5.67	0.00	1.68	0.00	0.80	1.05	2.06	0.27	2.41	0.31	2.04	7	−0.650	1.18709	4.94303	*PLTP*	chr20	2.86
NM_000582	12.85	0.00	10.11	0.15	1.92	0.00	0.62	0.46	0.25	0.77	22.29	0.00	0.82	0.00	12.40	0.44	4.60	0.46	8	0.650	7.31808	0.254295	*SPP1*	chr4	2.86
NM_138295	1.19	0.04	7.80	0.15	26.00	0.34	0.12	0.10	2.54	0.13	1.35	0.03	0.24	0.07	0.11	0.06	1.91	0.03	7	0.650	4.58405	0.105711	*PKD1L1*	chr7	2.85
NM_001113490	0.91	6.86	0.47	6.16	0.85	5.58	6.05	6.02	1.13	4.24	0.41	4.32	5.01	3.85	0.84	4.51	0.09	6.42	7	−0.650	1.7513	5.32956	*AMOT*	chrX	2.85
NM_001037160	0.42	4.07	0.22	3.97	0.21	3.47	3.94	5.35	1.04	1.35	0.13	1.15	2.20	1.83	0.00	1.68	0.13	1.24	6	−0.647	0.921785	2.67876	*CYS1*	chr2	2.83
NM_001164257	2.43	12.01	1.81	7.08	0.24	4.30	6.86	7.77	1.07	7.60	0.00	2.43	2.10	1.50	0.62	2.28	0.80	3.17	7	−0.647	1.76833	5.35072	*C17orf72*	chr17	2.83
NM_176813	0.96	10.55	0.57	10.19	0.00	8.48	7.16	5.94	1.09	3.65	0.00	11.01	2.46	4.38	3.99	3.47	0.00	3.70	6	−0.647	1.8038	6.81801	*AGR3*	chr7	2.83
NM_013280	6.97	0.21	14.89	0.27	4.66	0.23	0.25	0.11	1.40	0.07	5.78	0.04	0.96	0.22	0.32	0.00	5.05	0.16	9	0.646	4.47702	0.14489	*FLRT1*	chr11	2.83
NM_014643	2.19	8.24	2.18	6.77	0.40	5.77	8.28	8.26	2.96	8.45	0.28	8.46	5.56	7.28	2.72	7.07	0.08	10.32	7	−0.645	2.73999	7.84666	*ZNF516*	chr18	2.81
NM_012474	29.96	4.16	22.99	4.79	30.44	4.56	2.97	4.33	8.59	1.61	7.78	1.18	4.71	1.53	2.82	1.57	16.02	2.72	7	0.645	14.0308	2.93778	*UCK2*	chr1	2.81
NM_001170963	1.65	39.83	1.53	8.32	1.89	3.05	1.85	4.36	6.89	17.80	0.00	21.97	17.06	16.24	1.30	17.17	7.16	15.02	7	−0.643	4.37106	15.973	*IGSF1*	chrX	2.80
NM_003507	1.09	10.20	1.76	7.95	1.14	6.48	6.00	5.85	1.97	3.82	0.05	3.11	3.37	3.23	0.82	5.24	0.14	4.51	6	−0.642	1.81482	5.59805	*FZD7*	chr2	2.79
NM_000800	0.80	5.53	0.33	3.75	0.00	3.80	3.70	3.41	2.71	2.57	0.00	3.93	2.93	3.46	0.13	6.94	0.00	2.96	6	−0.639	1.17801	4.04017	*FGF1*	chr5	2.77
NM_002403	4.91	26.03	5.95	18.51	0.00	9.42	10.56	13.35	1.03	3.64	2.39	9.13	5.39	10.46	1.79	3.76	4.55	10.93	7	−0.639	4.06329	11.6929	*MFAP2*	chr1	2.77
NM_017711	0.67	5.73	0.00	3.13	0.38	4.72	3.23	3.33	0.80	0.92	0.00	1.45	0.75	1.55	0.12	1.48	0.00	2.19	7	−0.636	0.660344	2.72132	*GDPD2*	chrX	2.75
NM_182914	7.17	0.47	16.70	1.99	11.68	1.98	2.79	3.74	12.92	5.48	20.26	4.64	7.34	6.49	61.69	6.38	25.40	5.70	7	0.629	18.4396	4.09437	*SYNE2*	chr14	2.70
NM_014265	0.99	5.80	0.32	3.01	0.25	3.08	4.11	3.69	3.12	9.53	0.35	5.71	4.58	4.93	3.92	7.13	0.28	7.44	6	−0.629	1.99274	5.59059	*ADAM28*	chr8	2.70
NM_138568	1.61	6.24	1.00	8.24	0.52	4.33	4.73	3.77	1.57	4.01	0.48	3.10	3.38	3.24	1.25	4.23	0.06	2.71	7	−0.626	1.62213	4.43192	*EXOC3L2*	chr19	2.68
NM_001007097	1.91	4.88	0.31	3.59	0.61	4.25	5.84	5.59	1.30	2.70	0.42	5.29	4.96	5.14	0.38	2.35	0.20	5.39	7	−0.624	1.77023	4.3519	*NTRK2*	chr9	2.66
NM_000120	9.84	0.45	25.44	2.19	12.43	1.53	0.59	0.62	0.23	0.00	1.84	0.00	0.61	0.30	33.16	0.30	1.33	0.17	8	0.624	9.49584	0.616705	*EPHX1*	chr1	2.66
NM_024735	61.07	13.73	105.73	14.25	109.67	13.53	10.26	10.97	23.27	12.63	74.64	8.15	16.77	11.75	37.84	9.82	58.67	18.46	6	0.621	55.3231	12.5864	*FBXO31*	chr16	2.64
NM_031891	8.22	0.62	8.82	0.69	12.28	0.65	0.51	0.68	0.56	0.33	11.36	0.16	0.67	0.31	1.44	0.08	11.87	1.61	7	0.621	6.19342	0.569858	*CDH20*	chr18	2.64
NM_021168	15.71	2.25	6.52	2.22	9.00	1.47	2.86	1.00	8.99	3.29	7.51	3.10	2.97	3.02	14.77	2.89	7.49	3.31	8	0.621	8.42429	2.50629	*RAB40C*	chr16	2.64
NM_002501	5.22	14.86	1.57	11.02	0.84	13.40	12.84	7.77	5.96	13.77	2.08	15.85	9.25	14.48	2.00	12.13	1.37	13.95	7	−0.620	4.56958	13.0244	*NFIX*	chr19	2.63
NM_020848	1.11	2.67	0.47	5.85	0.37	2.25	3.72	3.17	1.56	4.88	0.95	5.86	5.12	4.39	0.86	4.08	0.39	5.48	7	−0.619	1.61734	4.29134	*KIAA1462*	chr10	2.63
NM_000915	11.05	29.14	1.18	58.76	0.13	1.62	35.57	24.18	1.81	2.93	0.23	8.23	1.24	0.47	0.00	3.65	0.00	2.10	7	−0.617	5.69113	14.5656	*OXT*	chr20	2.61
NM_021248	11.16	1.32	20.84	0.80	13.01	0.93	0.37	0.81	2.38	0.25	5.64	0.14	0.54	0.08	0.73	0.03	4.70	0.46	9	0.616	6.59668	0.537555	*CDH22*	chr20	2.60
NM_177963	1.05	5.32	0.29	4.14	0.22	4.16	4.19	2.45	1.96	3.20	0.22	6.38	4.59	4.34	0.40	5.73	0.04	5.35	6	−0.615	1.43936	4.56256	*SYT12*	chr11	2.60
NM_024081	0.55	5.05	0.64	2.75	1.12	4.51	4.00	3.53	1.67	5.12	1.30	4.35	2.98	3.77	0.31	3.27	0.69	3.32	7	−0.609	1.47354	3.9624	*PRRG4*	chr11	2.56
NM_003256	2.98	10.44	2.81	9.77	1.69	10.21	10.08	13.04	1.66	3.73	0.20	2.91	3.38	3.85	1.49	3.30	0.17	3.26	7	−0.607	2.71861	6.72398	*TIMP4*	chr3	2.55
NM_020546	22.43	3.63	22.42	4.16	21.52	3.41	3.94	3.53	4.68	2.13	11.58	2.51	3.98	3.35	27.52	4.16	18.35	3.90	7	0.607	15.1575	3.41924	*ADCY2*	chr5	2.54
NM_024572	1.06	5.70	0.73	2.56	0.50	4.00	3.45	1.57	2.06	5.65	0.00	3.77	1.99	2.86	0.15	3.12	0.00	4.94	8	−0.605	1.10593	3.79552	*GALNT14*	chr2	2.53
NM_032963	25.43	81.84	26.45	187.39	17.59	145.55	189.19	153.18	18.83	32.53	6.70	29.91	19.11	25.45	4.39	19.97	0.71	16.40	6	−0.602	34.266	76.913	*CCL14*	chr17	2.51
NM_015687	4.47	1.30	6.13	1.85	8.90	1.43	1.43	1.35	10.24	4.03	23.25	4.10	5.40	4.33	14.89	3.00	22.09	5.81	7	0.601	10.7563	3.02213	*FILIP1*	chr6	2.51

Table S10. ACTH highly related-genotypes.

Gene	FC	PCC	*p* Value	FDR
PPP1R1B	4.18	0.980	<0.001	0.001
TNFAIP8L3	3.12	0.961	<0.001	0.001
AHRR	2.65	0.950	<0.001	0.003
PNMT	3.51	0.944	<0.001	0.003
THBS2	2.52	0.939	<0.001	0.003
LRRC10B	2.73	0.938	<0.001	0.003
CREB3L1	3.82	0.931	<0.001	0.004
IER3	2.54	0.929	<0.001	0.004
GPR50	3.15	0.929	<0.001	0.003
FOSB	3.00	0.924	<0.001	0.003
TFPI2	3.27	0.922	<0.001	0.004
CELSR1	2.53	0.915	0.001	0.005
HMGN5	2.55	0.909	0.001	0.005
STRC	3.06	0.908	0.001	0.005
HHATL	3.12	0.901	0.001	0.005
AVPR1B	4.42	0.897	0.001	0.005
VSTM1	3.25	0.896	0.001	0.005
ENPP2	2.50	0.894	0.001	0.005
PDLIM4	3.25	0.893	0.001	0.005
ASAP2	3.41	0.893	0.001	0.004
PLEKHG5	3.12	0.893	0.001	0.004
UGT2A1	2.83	0.893	0.001	0.004
SOCS3	2.68	0.891	0.001	0.004
SLIT1	3.64	0.889	0.001	0.004
INMT	3.05	0.884	0.002	0.004
CHIA	2.84	0.878	0.002	0.004
MDFI	3.07	0.874	0.002	0.005
FAM92B	2.51	0.866	0.003	0.005
GHRHR	3.16	0.861	0.003	0.006
ABCB9	2.89	0.853	0.003	0.007
HTATSF1	2.99	0.853	0.003	0.007
MFAP4	3.35	0.850	0.004	0.007
C14orf132	2.58	0.849	0.004	0.007
ZNF331	2.92	0.848	0.004	0.007
PEBP4	2.54	0.847	0.004	0.007
GNAL	3.51	0.842	0.004	0.008
SHISA9	7.58	0.838	0.005	0.008
MYCL1	2.60	0.831	0.006	0.009
CACNA2D3	4.05	0.825	0.006	0.010
RASD1	3.34	0.824	0.006	0.010
GPR68	5.00	0.824	0.006	0.010
SHROOM2	2.81	0.820	0.007	0.011
ARC	2.97	0.820	0.007	0.011
B4GALNT4	2.53	0.818	0.007	0.011
VWA5B1	2.66	0.816	0.007	0.011
SCUBE1	3.25	0.790	0.011	0.016
AOC2	2.70	0.789	0.011	0.016
CA4	3.12	0.783	0.013	0.018

Table S10. *Cont.*

Gene	FC	PCC	*p* Value	FDR
COL9A3	2.78	0.782	0.013	0.018
FOLR1	3.01	0.777	0.014	0.019
ADCY5	2.62	0.768	0.016	0.021
UNC5A	4.04	0.758	0.018	0.023
CNTN2	4.19	0.757	0.018	0.023
SEP14	2.65	0.751	0.020	0.025
CCDC68	3.49	0.751	0.020	0.025
FRMD3	3.09	0.750	0.020	0.025
ASCL1	2.93	0.748	0.020	0.025
FRMD3	3.03	0.748	0.020	0.025
CNKSR1	2.51	0.736	0.024	0.028
CYR61	2.96	0.710	0.032	0.036
PRRG4	2.56	0.707	0.033	0.037
COL8A1	2.73	0.706	0.034	0.037
TBX20	3.83	0.699	0.036	0.039
GH2	4.57	0.689	0.040	0.042
RAMP1	2.53	0.678	0.045	0.047
FAM107B	2.55	0.674	0.047	0.047
LMX1A	3.94	0.674	0.047	0.047
MAMLD1	3.60	0.673	0.047	0.047
DRD2	2.64	0.670	0.048	0.048

Table S11. Cortisol highly related-genotypes.

Gene	FC	Blood F 8 AM	Blood F 0 AM	24 h UFC (Control)	24 h UFC (Control) Small Doses of the Second Day	24 h UFC (Control) High Doses of the Second Day
SHISA9	7.58	0.75	—	—	—	—
GPR50	3.15	0.74	0.98	—	—	—
PPP1R1B	4.18	0.71	0.88	—	—	—
HHATL	3.12	0.69	0.92	—	—	—
ASAP2	3.41	0.69	0.91	—	—	—
PLEKHG5	3.12	0.69	0.91	—	—	—
PDLIM4	3.25	0.69	0.91	—	—	—
UGT2A1	2.83	0.69	0.91	—	—	—
PNMT	3.51	0.68	0.90	—	—	—
ENPP2	2.50	0.68	0.92	—	—	—
AHRR	2.65	0.67	0.91	—	—	—
CHIA	2.84	—	0.97	—	—	—
TNFAIP8L3	3.12	—	0.93	—	—	—
LRRC10B	2.73	—	0.91	—	—	—
CREB3L1	3.82	—	0.93	—	—	—
CELSR1	2.53	—	0.88	—	—	—
STRC	3.06	—	0.95	—	—	—
VSTM1	3.25	—	—	0.72	—	—
SLIT1	3.64	—	0.89	—	—	—
INMT	3.05	—	0.89	—	—	—
GHRHR	3.16	—	0.92	—	—	—
ABCB9	2.89	—	0.91	—	—	—
MFAP4	3.35	—	0.93	—	—	—
C14orf132	2.58	—	0.89	—	—	—
SHROOM2	2.81	—	0.88	—	—	—
VWA5B1	2.66	—	0.89	—	—	—
AOC2	2.70	—	0.91	—	—	—
CA4	3.12	—	0.89	—	—	—

Table S11. *Cont.*

Gene	FC	Blood F 8 AM	Blood F 0 AM	24 h UFC (Control)	24 h UFC (Control) Small Doses of the Second Day	24 h UFC (Control) High Doses of the Second Day
CCDC68	3.49	—	0.91	—	—	—
FRMD3	3.03	—	0.92	—	—	—
CNKSR1	2.51	—	0.93	—	—	—
PRRG4	2.56	—	0.90	—	—	—
TFPI2	3.27	—	0.99	—	—	—
SCUBE1	3.25	—	0.98	—	—	—
CYFIP2	2.69	—	—	0.72	—	—
KIAA1024	5.94	—	0.00	—	0.76	—
RAB40C	2.64	—	0.00	—	0.91	0.73
ELMOD1	2.74	—	0.91	—	—	—
SCOC	3.96	—	0.91	—	—	—
PPP1R27	2.71	—	0.88	—	—	—
FBLN1	2.85	—	0.90	—	—	—
DTNA	4.99	—	—	0.79	—	—
ACAN	4.24	—	-0.90	—	—	—
CRHR1	4.10	—	—	—	0.75	0.78
MAMLD1	3.31	—	0.89	—	—	—
C3orf72	4.57	—	0.98	—	—	—
TNNT2	4.04	—	—	—	0.72	0.76
DIO2	3.92	—	—	0.68	—	—
NELL2	3.73	—	—	0.74	—	—
NCOA7	3.33	—	0.90	—	0.71	0.75
SKAP1	3.24	—	—	0.79	—	—
RIIAD1	3.08	—	—	0.69	—	—
MGP	3.08	—	0.88	—	—	—
C19orf33	2.95	—	—	0.68	—	—
SMAD3	2.81	—	—	0.79	—	—
MFAP2	2.77	—	—	0.79	—	—
S100A13	2.62	—	—	0.79	—	—
LSP1	2.60	—	0.89	—	—	—
PRR5	2.52	—	0.91	—	—	—
CPNE4	2.88	−0.69	—	—	—	—
SLC38A3	2.96	−0.69	—	—	—	—
CNGA3	2.67	−0.69	—	—	—	—
PPIF	2.58	−0.70	—	—	—	—
SCN1B	3.06	−0.71	—	—	—	—
FLRT1	2.83	−0.72	—	—	—	—
APOL4	2.97	−0.73	—	—	—	—
PPAPDC1A	2.82	−0.75	—	—	—	−0.71
OXT	2.61	−0.80	—	—	—	—

—: *p* value > 0.05.

Table S12. LH highly related-genotypes.

Gene	FC	PCC	*p* Value	FDR
POU1F1	6.26	0.922	<0.001	0.017
DRD2	2.85	0.898	0.001	0.021
LHB	2.64	0.895	0.001	0.015
ALDOB	2.73	0.886	0.001	0.015
TBX20	3.83	0.874	0.002	0.017
GH2	4.57	0.870	0.002	0.016
LOC100507588	3.38	0.865	0.003	0.015
GPR50	3.15	0.862	0.003	0.014
HSPB3	5.62	0.849	0.004	0.017
SEP14	2.65	0.829	0.006	0.023
APLN	2.59	0.816	0.007	0.027
DRD2	2.64	0.805	0.009	0.030
CNTN2	4.19	0.799	0.010	0.031
DLK1	4.87	0.797	0.010	0.029
HMP19	3.37	0.796	0.010	0.028
LYVE1	3.38	0.782	0.013	0.033
MAMLD1	3.60	0.773	0.014	0.035
MARCO	2.72	0.772	0.015	0.034
COL9A3	2.78	0.769	0.015	0.033
SCUBE1	3.25	0.753	0.019	0.039
GNA14	3.88	0.752	0.019	0.038
COL8A1	2.73	0.746	0.021	0.037
SERTM1	4.45	0.746	0.021	0.036
MEGF11	3.67	0.744	0.022	0.035
TFPI2	3.27	0.743	0.022	0.034
FLRT1	2.83	0.740	0.023	0.035
LMX1A	3.94	0.738	0.023	0.034
MDK	2.98	0.736	0.024	0.034
MYCL1	2.60	0.735	0.024	0.033
CHIA	2.84	0.732	0.025	0.033
ANKS1B	2.54	0.725	0.027	0.035
ZNF331	2.92	0.719	0.029	0.036
POU1F1	3.95	0.712	0.031	0.038
FAM92B	2.51	0.701	0.035	0.041
PMAIP1	4.66	0.692	0.039	0.044
GNAL	3.51	0.687	0.041	0.045
ARC	2.97	0.683	0.043	0.046
CYFIP2	3.22	0.668	0.049	0.052
RARA	3.51	0.668	0.049	0.050
CDH20	2.64	0.668	0.049	0.049
IL20RA	2.53	−0.750	0.020	0.037

Table S13. E2 highly related-genotypes.

Gene	FC	PCC	*p* Value	FDR
FXYD1	4.53	0.952	<0.001	0.001
CBFA2T2	3.25	0.940	<0.001	0.001
FSTL5	2.84	0.939	<0.001	0.001
CBS	4.15	0.934	<0.001	0.001
B3GAT1	2.95	0.934	<0.001	0.001
SEZ6L	3.88	0.934	<0.001	<0.001
FGF13	4.07	0.933	<0.001	<0.001
PRR5	2.58	0.722	0.028	0.030
FGF12	3.12	0.705	0.034	0.034

Table S14. Information of five selected genes.

ID	N1		N2		N3		N4		N5		N6		N7		N8		N9		Differentail Recurrence	DI	Avg (Tumor)	Avg (ANPTs)	Gene Symbol	Chr
	Tumor	ANPTs	Tumor	ANPTs	Tumor	ANPTs	Tumor	ANPTs	Tumor	ANPTs	Tumor	ANPTs	Tumor	ANPTs	Tumor	ANPTs	Tumor	ANPTs						
NM_001146	0.66	2.88	0.00	2.37	0.01	3.50	2.69	2.01	2.22	6.27	0.00	10.09	5.94	5.73	1.33	7.52	0.02	13.19	7	−0.693	1.431	5.951	*ANGPT1*	chr8
NM_000088	40.10	5.07	43.31	9.33	77.52	7.59	5.41	7.36	16.26	6.24	21.30	3.31	12.44	3.88	24.57	4.79	24.26	5.71	8	0.668	29.463	5.921	*COL1A1*	chr17
NM_002526	15.62	1.62	18.26	2.64	13.47	1.33	3.39	1.73	8.57	2.10	31.88	2.07	3.84	5.18	4.71	1.69	17.51	3.64	7	0.667	13.027	2.445	*NT5E*	chr6
NM_002775	19.77	134.27	11.63	98.22	9.45	70.44	87.43	111.71	38.47	79.78	8.71	108.97	68.77	97.49	10.90	121.24	9.96	68.46	7	−0.702	29.454	98.956	*HTRA1*	chr10
NM_001040058	39.45	1.44	37.05	0.95	7.99	1.54	3.06	0.85	1.41	3.42	87.76	0.54	0.00	0.93	33.11	2.06	11.36	1.19	9	0.634	24.577	1.437	*SPP1*	chr4

Table S15. Primer sequences.

Gene Symbol	Forward (5′ to 3′)	Reverse (5′ to 3′)
SPP1	GCATTCCGATGTGATTGAT	GTCTACAACCAGCATATCTTC
COL1A1	CACGACAAAGCAGAAACA	ATCAAGACAAGAACGAGGTA
NT5E	GATGGCTCCTCTCAATCA	TGTGTCTCAGGTTGTTGT
HTRA1	ACGGTGAAGTGATTGGAA	TGAGTGACATCATTCGGATA
ANGPT1	CCGAGCCTATTCACAGTAT	AATCAGCACCGTGTAAGA
GAPDH	GGTGGTCTCCTCTGACTTCAACA	GTTGCTGTAGCCAAATTCGTTGT

Table S16. Full name and unit of clinical indexes.

ID	Clinical Indexes	Full Name	Untis
	Tumor size		
1	Maximum diameter	Maximum diameter	mm
2	Gross tumor volume	Gross tumor volume	mm^3
	ACTH-secreting pituitary adenoma related hormones		
3	ACTH 8 AM	8 AM plasma adrenocorticotropic hormone level	pg/mL
4	8 AM F	8AM total serum cortisol level	ug/dL
5	0 AM F	Midnight AM total serum cortisol level	ug/dL
6	24 h UFC (control)	24-h Urinary-free cortisol test (control)	ug
7	24 h UFC (low-dose DST)	24-h Urinary cortisol level (low-dose dexamethasone suppression test)	ug
8	24 h UFC (high-dose DST)	24-h urinary cortisol level (high-dose dexamethasone suppression Test）	ug
	Other pituitary related hormones		
9	GH	Growth hormone	ng/mL
10	IGF-1	Insulinlike growth factor 1	ng/mL
11	LH	Luteinizing hormone	mIU/mL
12	FSH	Follicle-stimulating hormone	mIU/mL
13	E2	Estradiol	pg/mL
14	PRL	Prolactin	ng/mL
15	TSTO	Testosterone	ng/dL
16	PROG	Progesterone	ng/mL
17	FT3	Serum free triiodothyronine	pg/mL
18	FT4	Serum free thyroxine	ng/dL
19	T3	Triiodothyronine	ng/mL
20	T4	Thyroxine	ug/dL
21	TSH	Serum thyroid stimulating hormone	uIU/mL
	Bone metabolism		
22	PTH	Serum parathyroid hormone	pg/mL
23	P	Serum calcium	mmol/L
24	Ca	Serum phosphate	mmol/L
25	ALP	Serum alkaline phosphatase	U/L
	Age-matched bone mineral density (BMD)		
26	L1	First lumbar Z-score	score
27	L2	Second lumbar Z-score	score
28	L3	Third Lumbar Z-score	score
29	L4	Fourth Lumbar Z-score	score
30	L1-2	lumbar 1-2 Z-score	score
31	L1-4	lumbar 1-4 Z-score	score
32	L2-4	lumbar 2-4 Z-score	score
33	Neck of femur	Neck of femur Z-score	score
34	Greater trochanter	Greater trochanter Z-score	score
35	Total Haunch Bone	Total haunch bone Z-score	score
	Routine blood chemistry		
36	WAC	White blood cell	10^9/L
37	LY% (%)	Lymphocyte	%
38	MONO% (%)	Monocyte	%
39	NEUT% (%)	Ueutrophile granulocyte	%
40	EOS (%)	Eosnophils	%
41	BASO (%)	Basophilic granulocyte	%
42	LUC (%)	Large unstained cell	%
43	LY#	Lymphocyte	10^9/L
44	MONO#	Monocyte	10^9/L
45	NEUT#	Neutrophile granulocyte	10^9/L
46	EOS#	Eosnophils	10^9/L
47	BASO#	Basophilic granulocyte	10^9/L
48	RBC	Red blood cell	10^{12}/L
49	HGB	Hemoglobin	g/L

Table S16. *Cont.*

ID	Clinical Indexes	Full Name	Untis
50	HCT	Hematocrit	%
51	MCV	Mean corpuscular volume	fl
52	MCHC	Mean corpuscular hemoglobin concentration	g/L
53	MCH	Mean corpuscular hemoglobin	pg
54	CHCM	Cellular hemoglobin concentration mean	g/L
55	CH	Cellular hemoglobin	pg
56	RDW-C	Red cell distribution width	%
57	HDW	Hemoglobin distribution width	%
58	PLT	Platelets	10^9/L
59	PCT	Plateletcrit	%
60	PDW	Platelet distribution width	%
61	MPV	Mean platelet volume	fl
62	ESR	Erythrocyte sedimentation rate	mm/h
	Cardiovascular related indexes		
63	hsCRP	High sensitivity C reactive protein	mg/L
64	SBP	Systolic blood pressure	mmHg
65	DBP	Diastolic blood pressure	mmHg
	Liver function		
66	ALT	Alanine aminotransferase	U/L
67	TP	Total protein	g/L
68	Alb	Albumin	g/L
69	A/G	Albumin/globulin	ratio
70	Tbil	Total bilirubin	umol/L
71	Dbil	Direct bilirubin	umol/L
72	GGT	γ-glutamyl transpeptidase	U/L
73	AST	Aspartic acid transaminase	U/L
74	TBA	Total bile acids	umol/L
75	LD	Lactic dehydrogenase	U/L
76	ChE	Acetylcholine esterase	kU/L
77	ADA	Adenosine deaminase	U/L
78	PA	Prealbumin	mg/L
	Electrolyte		
79	K	Kalium	mmol/L
80	Na	Sodium	mmol/L
81	Cl	Chlorine	mmol/L
82	TCO_2	Total carbon dioxide	mmol/L
	Renal function		
83	Cr(E)	Creatinine	umol/L
84	Urea	Urea	mmol/L
85	Glu	Glutamic acid	mmol/L
86	UA	Uric acid	umol/L
	Lipid metabolism		
87	TC	Total cholesterol	mmol/L
88	TG	Triglyceride	mmol/L
89	HDL-C	High-density lipoproteincholesterol	mmol/L
90	LDL-C	Low-density lipoprotein cholesterol	mmol/L
91	ApoA1	Apolipoprotein A1	g/L
92	ApoB	Apolipoprotein B	g/L
93	Lp(a)	Lipoprotein	mg/L
94	FFA	Free fatty acid	umol/L
	Glycometabolism		
95	blood gloucose	Blood glucose	mmol/L
96	GLU 0 min	Glucose 0 min	mmol/L
97	INS 0 min	Insulin 0 min	uIU/mL
98	GLU 30 min	Glucose 30 min	mmol/L
99	INS 30 min	Insulin 30 min	uIU/mL
100	GLU 60 min	Glucose 60 min	mmol/L
101	INS 60 min	Insulin 60 min	uIU/mL
102	GLU 120 min	Glucose 120 min	mmol/L
103	INS 120 min	Insulin 120 min	uIU/mL
104	GLU 180 min	Glucose 180 min	mmol/L
105	INS 180 min	Insulin 180 min	uIU/mL
106	HbAlc	Glycosylated hemoglobin	%
